# A comprehensive exploration of the druggable conformational space of protein kinases using AI-predicted structures

**DOI:** 10.1371/journal.pcbi.1012302

**Published:** 2024-07-24

**Authors:** Noah B. Herrington, Yan Chak Li, David Stein, Gaurav Pandey, Avner Schlessinger

**Affiliations:** 1 Department of Pharmacological Sciences, Icahn School of Medicine at Mount Sinai, New York, New York, United States of America; 2 Department of Genetics and Genomic Sciences, Icahn School of Medicine at Mount Sinai, New York, New York, United States of America; 3 Department of Artificial Intelligence and Human Health, Icahn School of Medicine at Mount Sinai, New York, New York, United States of America; University at Buffalo - The State University of New York, UNITED STATES OF AMERICA

## Abstract

Protein kinase function and interactions with drugs are controlled in part by the movement of the DFG and ɑC-Helix motifs that are related to the catalytic activity of the kinase. Small molecule ligands elicit therapeutic effects with distinct selectivity profiles and residence times that often depend on the active or inactive kinase conformation(s) they bind. Modern AI-based structural modeling methods have the potential to expand upon the limited availability of experimentally determined kinase structures in inactive states. Here, we first explored the conformational space of kinases in the PDB and models generated by AlphaFold2 (AF2) and ESMFold, two prominent AI-based protein structure prediction methods. Our investigation of AF2’s ability to explore the conformational diversity of the kinome at various multiple sequence alignment (MSA) depths showed a bias within the predicted structures of kinases in DFG-in conformations, particularly those controlled by the DFG motif, based on their overabundance in the PDB. We demonstrate that predicting kinase structures using AF2 at lower MSA depths explored these alternative conformations more extensively, including identifying previously unobserved conformations for 398 kinases. Ligand enrichment analyses for 23 kinases showed that, on average, docked models distinguished between active molecules and decoys better than random (average AUC (avgAUC) of 64.58), but select models perform well (e.g., avgAUCs for PTK2 and JAK2 were 79.28 and 80.16, respectively). Further analysis explained the ligand enrichment discrepancy between low- and high-performing kinase models as binding site occlusions that would preclude docking. The overall results of our analyses suggested that, although AF2 explored previously uncharted regions of the kinase conformational space and select models exhibited enrichment scores suitable for rational drug discovery, rigorous refinement of AF2 models is likely still necessary for drug discovery campaigns.

## Author summary

Greater abundance of kinase structural data in inactive conformations, currently lacking in structural databases, would improve our understanding of how protein kinases function, and expand drug discovery and development for this important family of therapeutic targets. Modern approaches utilizing artificial intelligence and machine learning, like AlphaFold2 and ESMFold, have potential for efficiently capturing novel protein conformations. We provide evidence for a bias within AlphaFold2 and ESMFold to predict structures of kinases in their active states, similar to their overrepresentation in the PDB. We show that lowering the multiple sequence alignment depth used in the AlphaFold2 algorithm can help explore the kinase conformational space more broadly. Through a series of quantitative and visual analyses of the models, we also offer a critique of AlphaFold2-generated models, highlighting their potential utility in drug discovery, but also underscoring the need for further refinement to enhance their suitability for rational drug design. Furthermore, many of the high-quality and high-enriching models we make available may represent starting points for novel drug discovery campaigns.

## Introduction

Protein kinases are key regulators of cell signaling pathways via phosphorylation of substrates, and are involved in a variety of processes, such as cell proliferation, movement, and growth, as well as immunological responses [1–4]. Protein kinases have been prominent targets for drug discovery against a variety of diseases [5–7]. Currently, 80 inhibitors that primarily target two dozen different kinases are FDA-approved [8]. However, kinases are difficult to target selectively [9,10]. The ATP-binding site in these proteins, which most inhibitors bind, is highly conserved [11,12], and druggable allosteric sites can be difficult to identify and target. Additionally, kinase structures are highly flexible and adopt different conformations [13,14], particularly in the ATP-binding site, which includes the DFG (Asp-Phe-Gly) motif. The conformation of this motif, among others’, partially dictates the kinase’s ability to catalyze the transfer ATP’s phosphoryl group to a substrate. The ‘DFG-in’ conformation, a requirement for catalytic activity [15], facilitates ATP binding [16], whereas the catalytically inactive ‘DFG-out’ state, characterized by a roughly 180° ‘flip’ of the Asp and Phe residues, precludes this [13,14,17]. A caveat of these conformational changes is that kinase catalytic activity requires the activation loop preceded by the DFG motif to remain in an ‘extended’ conformation, while the inactive DFG-out conformation is marked by a ‘folded’ activation loop [15,18]. The conformational plasticity and activity of kinases is also characterized by the movement of the nearby ɑC-Helix, which can also adopt ‘in’ and ‘out’ states [19].

Conformations of kinases are of great importance for pharmacology and drug discovery [13,14,17]. Kinase inhibitors, including multiple prescription drugs, have been classified based on the conformation to which they bind, and different inhibitor *types* show different biochemical and pharmacological profiles [20]. For instance, Type-II inhibitors targeting the catalytically inactive ɑC-Helix-in / DFG-out (CIDO) conformation exhibit longer residence times compared to Type-I inhibitors, which target the generally active ɑC-Helix-in / DFG-in (CIDI) conformation [21,22]. Furthermore, both DFG-out and ɑC-Helix-out conformations have additional, nearby allosteric pockets that can be targeted for drug discovery (e.g., ‘DFG’ or ‘back’ pocket) [23–27]. Recently, additional conformations have been described, which represent intermediate states that may enable the development of a unique class of kinase inhibitors [15,28]. The design of small molecule kinase inhibitors is aided by structural data in specific conformations they are intended to bind [29].

A major limitation in the development of conformation-specific kinase inhibitors (e.g., Type-II) is the relatively small number of structures in inactive states in the PDB [30] available for rational drug design [28,31]. Computational modeling has helped researchers bridge the gap in our structural knowledge of kinase conformations for drug design. For example, homology modeling- [32] and molecular dynamics (MD) simulations- [33] based methods have been applied to visualize alternative kinase conformations and advance the design of compounds targeting them [34,35]. However, homology modeling does not accurately model long regions that are unaligned to template structures [36]. Similarly, MD simulations often fail to sample the pharmacologically relevant movements (e.g. ‘DFG flip’) due to timescale and forcefield limitations, which limit their utility in kinase drug discovery [37].

Emerging *ab initio* prediction tools address some of these barriers by employing artificial intelligence (AI)-based modeling methods, which are advanced algorithms used to predict structures or generate 3D models with accuracies comparable to experimentally determined structures [38–41]. For example, AlphaFold2 (AF2) [40] and RosettaFold [38], use a multiple sequence alignment (MSA) to capture conserved contacts between evolutionarily related sequences needed to preserve the overall fold of the protein [42], as input. These learned contacts are then used within a deep learning framework to predict protein structures. Another example is ESMFold [41], which is an alignment-free, language-based model capable of predicting protein structures based on biological properties learned directly from sequence data.

Recent work has demonstrated that the modulation of some of the AF2 input parameters, such as MSA depth, may help explore structural diversity in membrane proteins [43], multidomain electron transfer complexes [44], and protein kinases [45–49]. Additional work has further examined the reliability of protein models generated by AF2 [45,50–52]. In this work, we performed the most comprehensive analysis to date of two representative AI-based structural modeling methods, i.e., AF2 and ESMFold, to explore the kinase conformational landscape relevant to drug discovery. Also, for the first time, we evaluated the predicted models in diverse structural conformations with respect to model quality (i.e., with pLDDT (“predicted local distance difference test”) score) [40], similarity to known experimental data (i.e., with TM (“template modeling”)-Score) [53], their ability to enrich for known ligands, and, by extension, their need for refinement prior to use in drug design. To assess possible reasons for this need, we inspected the binding sites of select models to assess their utility for rational ligand design by analyzing kinase-specific binding site obstructions, as well as the degree of movement of the activation loop frequently seen in DFG-out structures. Finally, based on our observations, we comment on various aspects of AF2’s usefulness in kinase drug design.

## Results

### Lowering MSA depth diminished the bias of AI prediction methods favoring particular conformations

**Fig 1:**
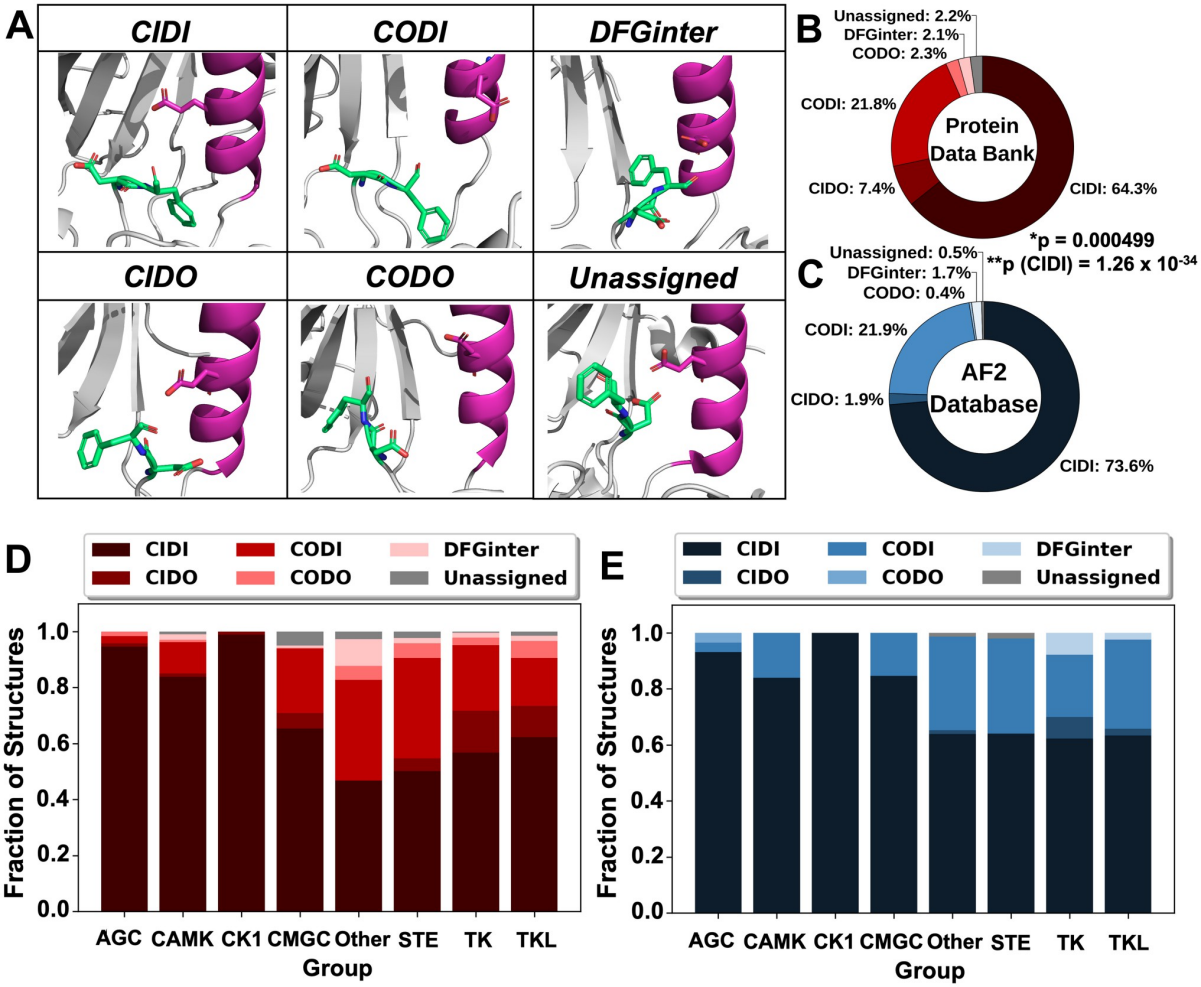
Kinase conformations in the PDB and AlphaFold2 (AF2) Protein Structure Database. (A) Prototypical structural conformations considered in our study: CIDI: ɑC-Helix/DFG-in; CIDO: ɑC-Helix-in/DFG-out; CODI: ɑC-Helix-out/DFG-in; CODO: ɑC-Helix-out/DFG-out; DFGinter (in between DFG-in and DFG-out); Unassigned (none-of-the-above). Examples shown are CIDI: MAPK14 (PDB ID: 1BL6), CIDO: RIPK2 (PDB ID: 4C8B), CODI: CDK2 (PDB ID: 1H07), CODO: NTRK1 (PDB ID: 6D1Y), DFGinter: AURKA (PDB ID: 3FDN), Unassigned: PDGFRA (PDB ID: 6JOJ). Residues in lime green indicate the aspartic acid and phenylalanine (DF) of the DFG motif, while magenta indicates the ɑC-Helix, whose conformation is signaled by movement of the conserved Glu residue. Red and blue atoms indicate oxygen and nitrogen atoms, respectively. (B) Fractional distribution of kinase structures from the PDB (n = 5,024) classified into each conformation type. (C) Fractional distribution of kinase models from the AF2 Database (n = 469) classified by conformation. *p-value was calculated using the Fisher’s exact test and indicated that the PDB and AF2 distributions were significantly different. **p(CIDI) was calculated using a one-sided Wilcoxon rank-sum and indicated AF2 had a significantly higher overrepresentation of CIDI models compared to the PDB. (D) Fractional distributions of PDB structures in different conformations by kinase group (AGC: PKA, PKG, PKC families; CAMK: Calcium/calmodulin-dependent; CK1: Casein kinase 1; CMGC: CDK, MAPK, GSK3, CLK families; STE: Sterile 7, Sterile 11, Sterile 20 kinases; TK: Tyrosine kinase; Tyrosine kinase-like). (E) Fractional distributions of models in different conformations by kinase group in the AF2 Database.

We first compared the current conformational space of experimentally determined structures of kinases in the PDB and structural models deposited in the AlphaFold2 Protein Structure Database (‘AF2 Database’) [54] and generated by ESMFold [41] (S1 Fig). In brief, we downloaded all available experimentally determined structures of 497 human kinase domains (from 484 kinases) from the PDB (5,136 structures for 331 proteins). While 153 kinases (32% of the kinome) had no known experimentally determined structure, each kinase of the 331 proteins with at least one known structure had over 15 structures on average. These structures were classifiable [55] into one of six defined conformations, including ɑC-Helix-in / DFG-in (CIDI), ɑC-Helix-in / DFG-out (CIDO), ɑC-Helix-out / DFG-in (CODI), ɑC-Helix-out / DFG-out (CODO), DFGinter (an in-between DFG conformation), and Unassigned (none-of-the-above) (Fig 1A). Of the classifiable structures, 50.0% only exhibited one conformation, highlighting the limited coverage of kinase conformations.

We also downloaded all computational models of these 497 kinase domains from the AF2 Database and predicted structures of the same domains using ESMFold, which were all also classified by their conformation. We calculated the fractional distribution of the six conformation types present in the PDB and AF2 Database (Fig 1B and 1C) and models predicted by ESMFold (S2 Fig). Our analysis revealed a significant overrepresentation of the active ‘CIDI’ state in all three datasets. Interestingly, both AF2 and ESMFold exhibited an even greater fraction of CIDI models than those in the PDB (73.6%, 82.9% and 65.7%, respectively); p(PDB_CIDI_ < AF2_CIDI_) = 1.26 x 10^−34^; p(PDB_CIDI_ < ESMFold_CIDI_) < 2.2 x 10^−16^. Furthermore, DFG-out conformations were underrepresented in all three datasets, more so in the AF2 Database and by ESMFold than in the PDB (2.3%, 0.8% and 9.7%, respectively; p(PDB_DFG-out_ > AF2_DFG-out_) < 2.2 x 10^−16^; p(PDB_DFG-out_ > ESMFold_DFG-out_) < 2.2 x 10^−16^). To test whether these differences arose from the expanded coverage of the kinome by AF2 or ESMFold, we limited analysis to kinase models with deposited structures in the PDB (n = 331) (“i.e., Overlap”). The distributions of AF2 and ESMFold kinase conformations showed similar trends when compared to their full datasets (S3A and S3B Fig, respectively; p(AF2 Overlap) = 0.751; p(ESMFold Overlap) = 0.913). This suggests that these *ab initio* predictors exhibit a bias for predicting structures of kinases in the active (CIDI) state.

Next, we investigated if the bias for the CIDI conformation existed within individual kinase family or evolutionary groups. We therefore computed the fractional distributions of each conformation by kinase group (AGC, CAMK, CK1, CMGC, Other, RGC, STE, TK and TKL) [56] using our datasets from the PDB and AF2 Database (Fig 1D and 1E) and the models predicted by ESMFold. We observed that the active conformation, CIDI, consistently represented the greatest fraction in all kinase groups in all three datasets, ranging from 46.7% (Other) to 98.9% (CK1) in the PDB, 62.2% (TK) to 100% (CK1) in the AF2 Database, and 58% (STE) to 100% (CK1 and CMGC) by ESMFold (S4 Fig). These same groups did not retain those similar distributions in ESMFold-predicted models (S4 Fig). Interestingly, the two groups with the highest fractions of CIDO structures in the PDB, TK and TKL, had lower CIDO fractions by AF2 and ESMFold. However, it is noteworthy that TK had the highest CIDO fraction in all three datasets. Intriguingly, the understudied CODO conformation was a minor population across all groups in the PDB, ranging from 0.0% (CK1) to 6.1% (TKL), but was even less frequent in the AI-predicted datasets, ranging from 0.0% (all but AGC) to 3.4% (AGC) for AF2 and 0.0% (all but AGC) to 1.7% (AGC) for ESMFold. Finally, in addition to the sole CODO model belonging to AGC generated by ESMFold, the only two Unassigned models belonged to the Other group, and the only three CIDO models belonged to TK. Taken together, our results suggest that the conformation bias seen across the kinome in the PDB (Fig 1B) was also observed at the group level for the PDB and both predictive methods, and that some groups are more conformationally diverse than others.

**Fig 2:**
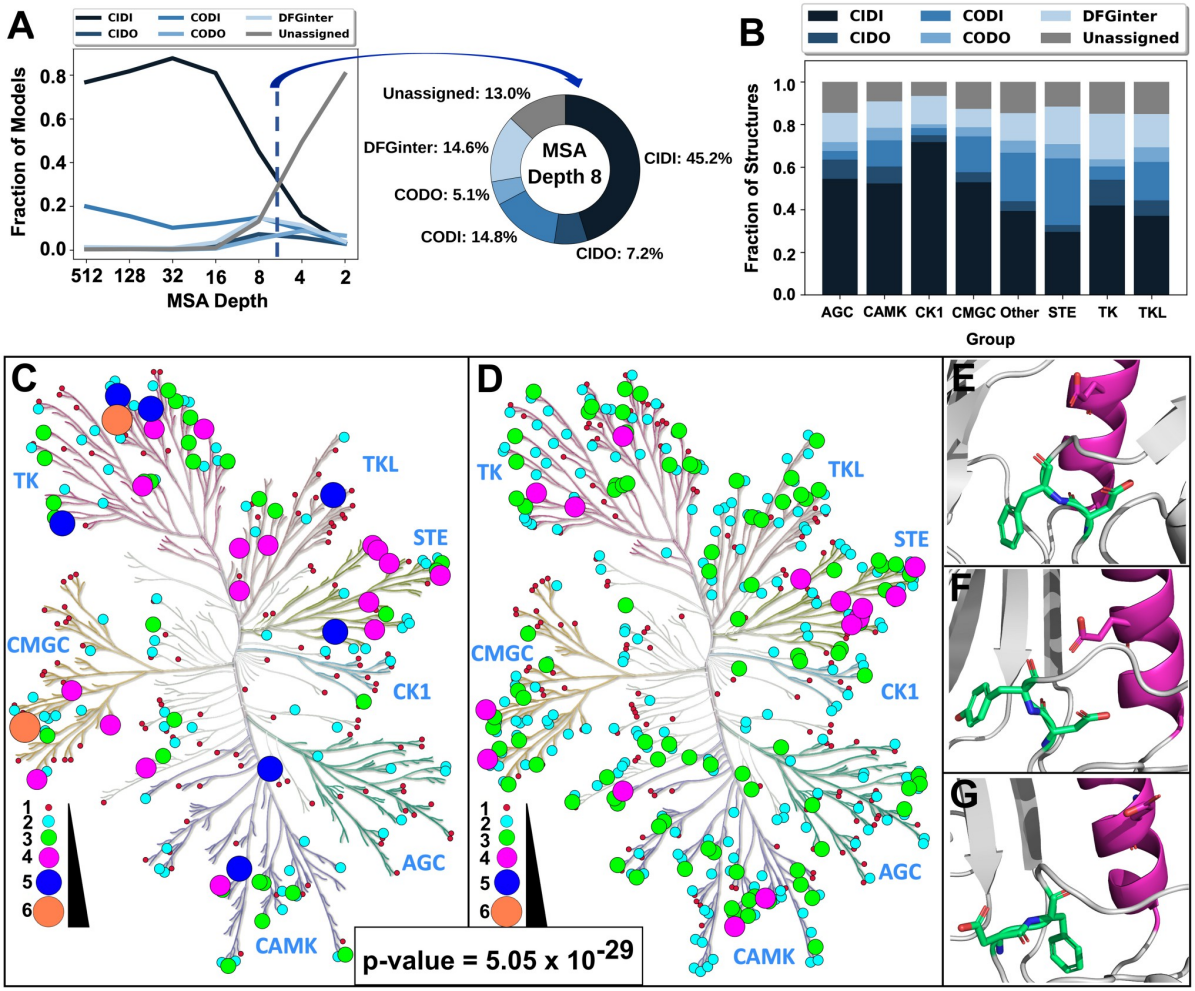
Exploration of the kinome’s conformational space by lowering MSA depth when predicting structures using AF2. (A) Fractional distribution of models in various conformations across different MSA depths (left plot) and at an MSA depth of 8 (donut plot). (B) Distributions of models predicted at an MSA depth of 8 by kinase group. (C) Coverage and conformational space representation across the human kinome tree, generated with KinMap, for structures from the PDB, where each node represents one kinase. Colors (crimson, cyan, lime, magenta, blue and coral, in order) and increasing circle sizes indicate greater counts of unique conformations. (D) Coverage and conformational space representation of the human kinome using models predicted by AF2 at an MSA depth of 8. The node color and size coding are the same as in C. (E-G) Representative models predicted by AF2 at an MSA depth of 8 for kinases from different groups in novel conformations not seen in the PDB: (E) CLK3 in the CIDO conformation, (F) NEK9 in the CIDO conformation, and (G) MAP3K4 in the CODI conformation. Residues in lime green represent the ‘DF’ of the DFG motif (‘DY’ of DYG for NEK9), while residues in magenta represent the ɑC-Helix. Red and blue atoms indicate oxygen and nitrogen atoms, respectively.

The AF2 algorithm includes a variety of parameters whose tuning can impact the predicted protein structural models. In particular, the number of sequences (‘depth’) in the multiple sequence alignment (MSA) used as input for AF2 correlates with the conformational diversity of the structural models generated of a given sequence [43], since the MSA generated by AF2 includes sequences evolutionarily related to the input kinase sequence. Therefore, to sample alternative kinase conformations, we used ColabFold [57] to run AF2 with various MSA depths as input (Methods). We generated five models for each kinase (default parameters), which were then classified into the same six conformations as earlier. We observed limited structural diversity in models generated at higher MSA depths of 512, 128, and 32, where most distributions appeared similar to those observed in the AF2 dataset, though only the distribution at a depth of 512 was statistically similar (Fig 2A; S1 Table). For example, at an MSA depth of 512, 76.8% of the models were in the CIDI conformation, while DFG-out states (CIDO and CODO) were under-represented (total of 1.7%).

Interestingly, we observed greater fractions of non-CIDI conformations in models generated at shallow MSAs (i.e., MSAs with fewer sequences) (Fig 2A). For instance, at an MSA depth of 8, the CIDI fraction of models was 45.2%, while the DFG-out states’ (CIDO and CODO) fraction was 12.3%. At lower MSA depths of 4 and 2, the models were even more diverse, respectively including only 15.7% and 3.2% of the models in the CIDI conformation. Furthermore, substantial fractions of the models (49.3% and 80.5%, respectively) were classified as Unassigned at these MSA depths. Statistical comparison of the fractional compositions of each conformation at each MSA depth to each other showed that they were all statistically different, but distributions at higher MSA depths (512 through 16) were more similar to that of the AF2 Database, while those of lower depths showed greater statistical difference from the AF2 Database (S2 Table). We attempted to enhance inactive conformation predictions (i) using custom MSAs and (ii) increasing the number of random seeds in as input to AF2: p(AF2 Database = MSA 256_32seeds) = 1; p(MSA 8_1seed = MSA 8_32seeds) = 1. However, these models exhibited similar conformational distributions to that observed in the AF2 Database (S5 Fig) (Methods).

Additionally, we observed that lowering MSA depth impacted the distribution of model conformations by kinase group (Fig 2B). First, the CIDI fraction at an MSA depth of 8 was much lower for all groups than the corresponding fractions in the AF2 Database and ranged from 29.6% (STE) to 71.7% (CK1) (Figs 1E and 2B). Second, the fractions of CIDO models in most groups was higher at an MSA depth of 8 (3.3% (CK1) to 12.1% (TK)) than the corresponding fractions in the AF2 Database (0.0% (many) to 7.8% (TK)). A similar trend was observed in CODO models for all groups (range of 1.7% (CK1) to 6.8% (STE) at an MSA depth of 8), except for AGC. The levels of statistical significance of all these comparisons are provided in the S3 Table.

To better understand these findings, we visualized the number of unique conformations for each kinase using KinMap [58], a tool for presenting data across the kinome, among PDB structures and AF2 (MSA depth = 8) models (Fig 2C and 2D, respectively). A comparison between these kinome trees demonstrated that lowering the MSA depth explored the conformational space of the kinome more broadly than the PDB and that, on average, an MSA depth of 8 yielded more conformations predicted per kinase over those with structures in the PDB. (p-value = 5.05 x 10^−29^). Part of this expansion were conformations not previously reported for several kinases. For instance, CLK3, NEK9, and MAP3K4, all from different kinase groups, had no experimentally determined structures in the PDB, but were predicted confidently in previously uncharacterized conformations (CIDI, CIDO, and CODI (CLK3); CIDI and CIDO (NEK9); CIDI, CODI, CODO, and DFGinter (MAP3K4)) by AF2. Collectively, the above data suggested that conformational exploration owing to different AF2 parameters is not limited to any one kinase group.

### Models in diverse and unseen conformations generated by AF2 at different MSAs varied in quality

**Fig 3:**
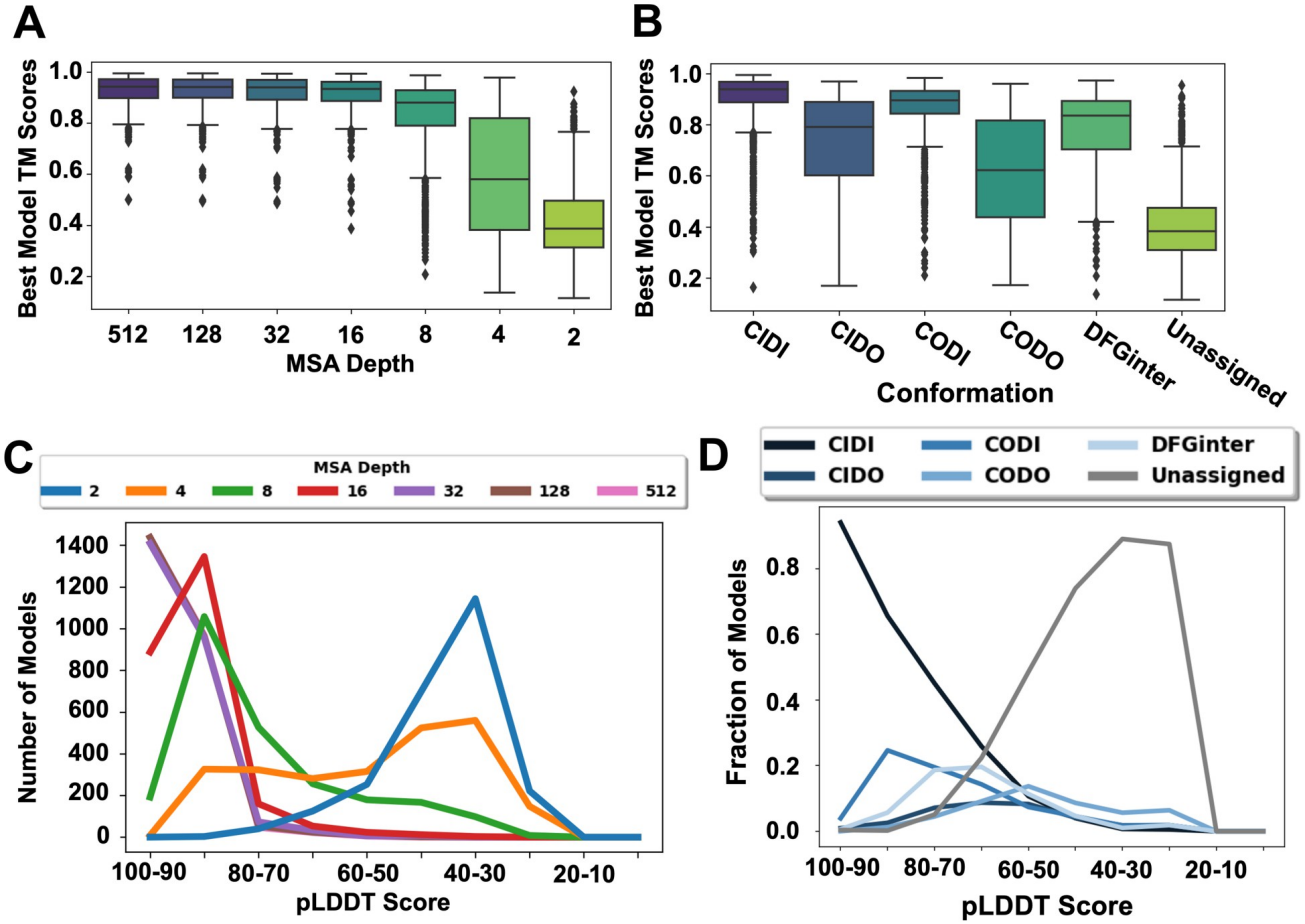
Quality of AF2 models by MSA and conformation type. Box (TM-Score) and line (pLDDT) plots summarizing the accuracy of kinase AF2 models, where TM-Scores measure similarity to experimental structures and pLDDT represents AF2’s confidence in its predictions. (A) Distributions of the highest TM-Scores calculated from comparisons of all PDB structures to all AF2 models of the same kinase by MSA depth (n = 1,145 scores for all depths). A trend of decreasing model accuracy is evident, especially from an MSA depth of 16 through 2. (B) Distributions of the best TM-Scores calculated from comparisons of all PDB structures to all AF2 models of the same kinase by conformation type (n = 4,692 (CIDI), 188 (CIDO), 908 (CODI), 246 (CODO), 405 (DFGinter), and 1,575 (Unassigned)). (C) Distribution of kinase models in terms of pLDDT across different MSA depths. High MSA depths typically yielded more confident models than lower depths. (D) Distribution of pLDDT scores of all models predicted across all MSA depths, grouped by conformation type.

While the above results demonstrated that AF2 can generate previously unseen kinase conformations, an assessment of model quality is a prerequisite for their utility for drug discovery and other applications. To evaluate the models’ quality, we compared them to experimentally determined structures from the PDB using the template modeling-score (TM-Score) [53], which is commonly used to evaluate protein structure prediction methods [40,59] (Methods). Briefly, we aligned each PDB structure with every AF2-predicted structure of the same kinase and calculated a TM-Score of the aligned structures (Fig 3A and 3B). We observed that the TM-Scores were generally lower at MSA depths of 8 (median = 0.88) and below (e.g., median = 0.38 at MSA depth 2) (Fig 3A). The outliers in these distributions largely resulted from highly flexible regions in the kinase domain that are frequently unstructured in PDB structures [60], predictions below AF2’s pLDDT threshold of confidence (i.e., < 70), or both. These results indicated that, while lower MSA depths did explore a larger conformational space, they were also likely to lead to models of lower accuracy. In terms of individual structural conformations, we observed that DFG-in models were more accurate than those in DFG-out and DFGinter conformations (Fig 3B), while DFG-out models (CIDO and CODO) tended to be more dissimilar to PDB structures.

One limitation of comparing AF2 models to PDB structures using similarity metrics like TM-score is that they penalize previously unobserved conformations that did not appear in the PDB. Therefore, we also calculated and analyzed the predicted local-distance difference test (pLDDT) scores [40,54] for the AF2-generated kinase models. The pLDDT score assesses the overall confidence in AF2’s prediction on a per-residue level, ranging from 0 (low confidence) to 100 (high confidence), and is more lenient toward structural novelty. We analyzed the distribution of average pLDDT scores taken from all residues in each model by MSA depth and conformation (Fig 3C and 3D). Our analysis revealed that, as the MSA depth decreased from 512 to 2, there was a nearly consistent drop in the overall confidence of models (Fig 3C). Models in the CIDI conformation were predicted with the highest level of confidence (94% at 90 < pLDDT < 100), while other conformations were more broadly distributed across the pLDDT range. Unassigned models were mostly predicted at low levels of confidence (89% at 30 < pLDDT < 40). Collectively, these data suggested that, while AF2 did produce models of varying accuracies in different conformations, excessive lowering of the MSA depth diminished the overall quality of predicted models. The conformational flexibility of the DFG motif in models predicted at different MSA depths is described in S1 Text and visualized in S6 Fig.

Encouraged by AF2’s ability to predict inactive conformations of kinases, we investigated whether if any kinases structures we predicted with AF2 were in previously unobserved conformations. For this, we compared all confident models (pLDDT > 70) generated by AF2 (Table 1) to PDB structures of the same kinases and calculated the number of models predicted in conformations not yet experimentally observed for their respective kinases at each MSA depth, including those kinases without a known structure in the PDB. A total of 6,297 models were confidently predicted in previously unobserved conformations, covering 398 kinases and 37% of all models generated across all MSA depths. This illustrates AF2’s ability to capture unexplored conformational spaces for many established and emerging drug targets (Fig 2C and 2D).

**Table 1:** Counts of confidently predicted AF2 models in conformations (columns) not seen for the same kinases in the PDB at multiple MSA depths (rows). Unique kinases across all MSA depths = 398 (185)^a^. pLDDT > 70 (pLDDT > 90)^b^.

MSA^c^	Kinases^d^	All^e^	CIDI	CIDO	CODI	CODO	DFGinter	Unassigned
**2**	**25 (0)**	**30 (0)**	**6 (0)**	**3 (0)**	**7 (0)**	**2 (0)**	**11 (0)**	**1 (0)**
**4**	**275 (2)**	**433 (2)**	**119 (0)**	**33 (0)**	**88 (0)**	**44 (1)**	**122 (1)**	**27 (0)**
**8**	**354 (68)**	**1066 (94)**	**480 (90)**	**98 (1)**	**180 (3)**	**49 (0)**	**236 (0)**	**23 (0)**
**16**	**278 (127)**	**1220 (406)**	**933 (395)**	**31 (0)**	**169 (11)**	**11 (0)**	**67 (0)**	**9 (0)**
**32**	**244 (153)**	**1197 (645)**	**1008 (620)**	**20 (4)**	**147 (16)**	**0 (0)**	**14 (1)**	**8 (4)**
**128**	**239 (160)**	**1178 (653)**	**918 (589)**	**17 (17)**	**215 (29)**	**1 (0)**	**18 (9)**	**9 (0)**
**512**	**239 (153)**	**1173 (645)**	**838 (557)**	**22 (21)**	**285 (48)**	**4 (0)**	**18 (13)**	**6 (6)**

^a^ ‘Unique kinases across all MSA depths’ signifies the total count of all sequence-unique kinases predicted by AF2 in novel conformations across all MSA depths.

^b^ Numbers given in parentheses represent the same counts for models with pLDDT scores higher than 90 to assess the robustness of the findings for even higher accuracy models.

^c^ ‘MSA’ indicates the MSA depth or the number of sequences used as input to AF2 to predict structural models of kinases.

^d^ ‘Kinases’ corresponds to the number of unique kinases predicted in novel conformations at each MSA depth.

^e^ ‘All’ corresponds to the total count of models predicted in novel conformations for their respective kinases at each MSA depth.

### AF2 kinase models exhibited varying levels of ligand enrichment

One limitation of performance measures such as pLDDT and TM-Score is that they measure overall quality of the model and provide little assessment of the binding site accuracy, an important consideration when using AF2 models in drug design. One empirical approach to evaluate the relevance of binding sites for drug discovery is through ligand enrichment, which assesses the models’ ability to dock known ligands more favorably than their decoys [61,62]. We calculated this enrichment for all confident AF2 models (pLDDT > 70) of a benchmark set of 23 kinases [62] using known ligands from the ChEMBL database [63] and decoys generated using the Directory of Useful Decoys-Enhanced (DUD-E) webserver [62] (Methods). After clustering known ligands, 50 decoys were generated for the 50 cluster center ligands for each of the 23 kinases to create chemically unique compound datasets that were docked against all models of their respective targets using Glide [64]. Finally, we generated enrichment curves and calculated the area under the curve (AUC) for each model.

**Fig 4:**
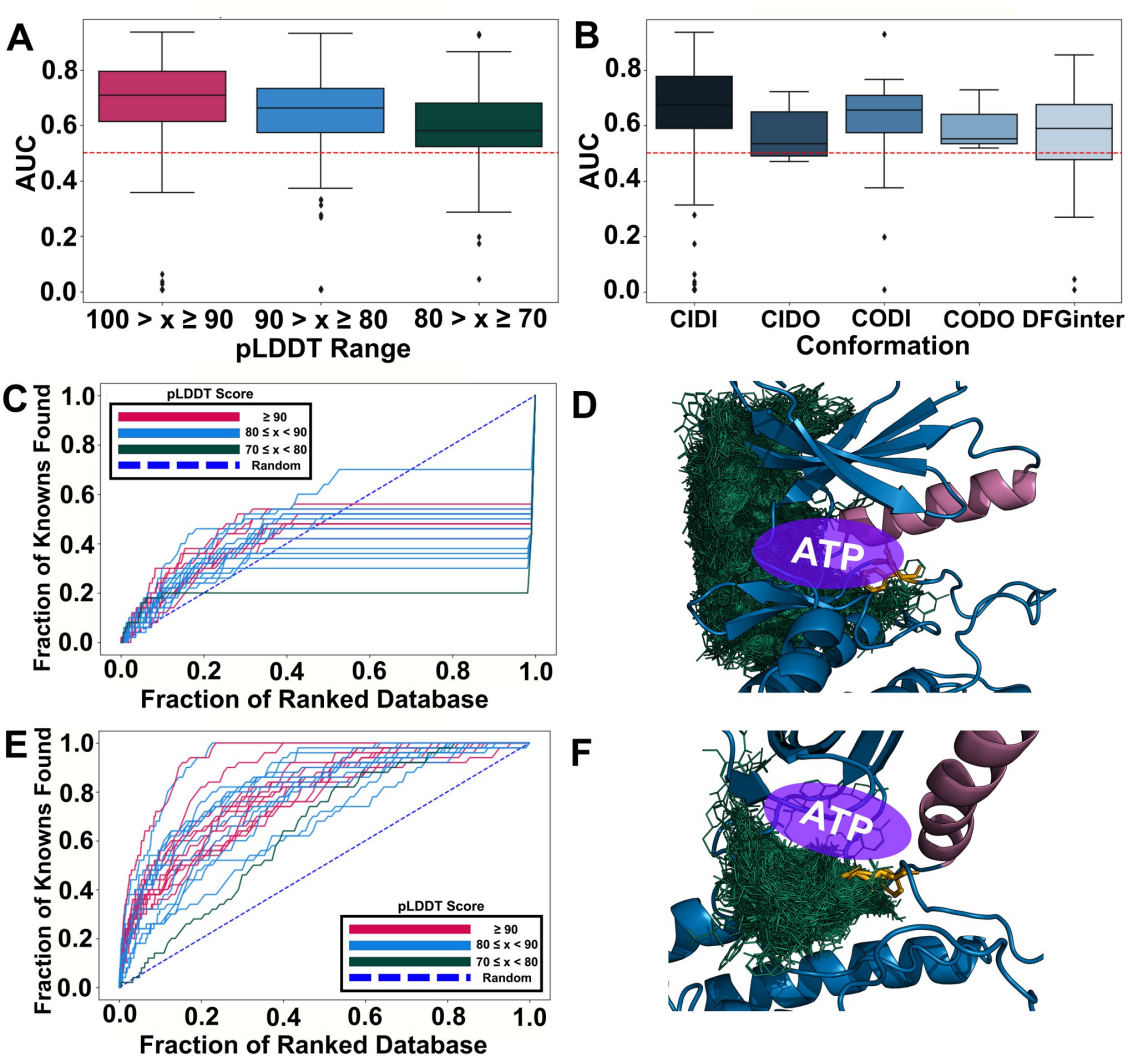
Enrichment analysis of protein kinase AF2 models. (A) Box plot showing the distributions of enrichment AUCs of confident AF2 models (pLDDT > 70) of 23 kinases. A dotted, red line indicates random enrichment, where models would be incapable of distinguishing known ligands from decoys. (B) Box plot showing the distributions of enrichment AUCs of AF2 models of 23 kinases by conformation type. (C) Enrichment curves of AF2 models of MAPK14 (avgAUC = 39.48, s.d. = 6.89). The blue dotted line represents random enrichment (AUC = 50). (D) Highest-enriching model of MAPK14 (maxAUC = 53.80). (E) Enrichment curves of AF2 models of PTK2 (avgAUC = 79.28, s.d. = 6.98). (F) Highest-enriching model of PTK2 (maxAUC = 93.67).

On average, we observed that the confident AF2 kinase models performed slightly, but significantly, better than random selection of ligands among decoys (p < 0.05). The average AUC for all enrichment curves for confident models (pLDDT ≥ 70) was 64.58 (standard deviation (s.d.) 17.27), indicating better than random selection of known ligands versus decoys. Additionally, more confident models by pLDDT yielded better enrichment, where models with pLDDT scores in ranges 100 > x ≥ 90, 90 > x ≥ 80, and 80 > x ≥ 70 had average AUCs of 67.50 (s.d. 18.56), 63.59 (s.d. 16.26), and 59.71 (s.d. 15.51), respectively (Fig 4A). Furthermore, all conformations but CODO (too few models) exhibited significantly better-than-random enrichment (Fig 4B). To assess possible reasons for differences in enrichment, we inspected some examples of weakly and highly enriching models. MAPK14 enriched poorly with an average AUC (avgAUC) of 39.48 (s.d. 6.89) and an AUC of 53.80 for the most enriching model (maxAUC) (Fig 4C; S4 Table). The vast majority of ligands were unable to dock to either the ATP site or core, but instead docked to the exterior of the kinase (Fig 4D). Further analysis revealed that the core was largely obstructed, thereby inhibiting docking of molecules to this site. In contrast, the models of PTK2 had some of the highest enriching models (avgAUC = 79.28, s.d. = 6.98) (Fig 4E). This kinase’s top-scoring model (maxAUC = 93.67) exhibited docking much closer to the ATP binding site and further into the core, suggesting that access to this site is required for adequate enrichment. (Fig 4F). A number of factors may contribute to ligand displacement in docking against kinases, as examined below.

**Fig 5:**
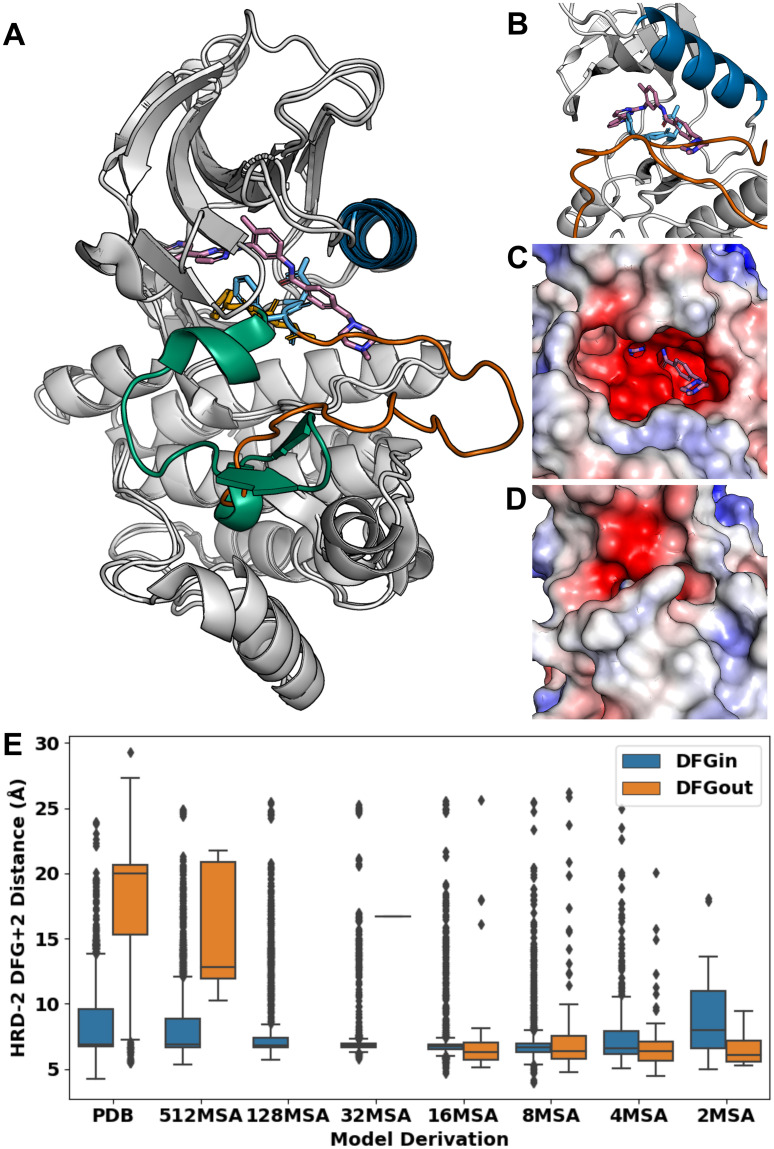
Examples of binding site occlusions by the DFG motif or activation loop in AF2 kinase models. (A) An AF2-predicted model of ABL1 at an MSA depth of 4 (pLDDT = 87.08) aligned to a crystal structure of ABL1 (PDB ID: 2HYY) complexed with imatinib. The ɑC helices of both models are colored in blue, the activation loops of the AF2 model and crystal structure are colored in burnt orange and green, respectively, the DFG motifs of the AF2 model and crystal structure are colored in sky blue and orange, respectively, and imatinib is colored in magenta. The Asp of the DFG motif in the ABL1 model swings upward to block access to the DFG pocket, a common observation made of many of our generated AF2 DFG-out models. (B) A close-up view of the DFG pocket of the AF2 model of ABL1. Both the upward-oriented Asp of the AF2 model’s DFG motif and its activation loop sterically clash with imatinib at its urea and piperazine moieties, respectively. Panels C and D offer the same perspective of the DFG pocket. (C) Electrostatic map representation of ABL1’s co-crystal structure with imatinib from the PDB. ABL1’s activation loop in its ‘folded’ conformation creates no obstruction to imatinib binding to the DFG pocket. (D) Electrostatic potential map of ABL1’s AF2 model of in a DFG-out state. The ‘extended’ activation loop partially occupies the DFG pocket and blocks access to it by imatinib’s piperazine moiety. (E) Box plot distributions of the HRD-2 and DFG+2 residue distances, signifying movement of the activation loop, in DFG-in versus DFG-out PDB structures and models confidently predicted by AF2. Models in both DFG-in and DFG-out states mostly have ‘extended’ activation loops, even at lower MSA depths, which promote conformational exploration. Frequent outliers result from tight distributions; the PDB contains many DFG-in structures with ‘folded’ activation loops, which is also true for DFG-in AF2 models.

A key challenge in kinase drug discovery is targeting the DFG-out (e.g., CIDO) states with conformation-specific inhibitors (e.g., Type-II) [20]. For example, ABL1 was predicted by AF2 in a DFG-out state and examined for its known ability to bind the prototypical Type-II inhibitor imatinib (IC_50_ = 1.1 nM) [65] (Fig 5). First, we superimposed a crystal structure of ABL1 with imatinib [66], with a CIDO model of the kinase generated by AF2 at an MSA depth of 4 (pLDDT = 87.08; RMSD = 0.783; Fig 5A). This comparison revealed a clear steric hindrance in the ABL1 model from the aspartic acid of the (D)FG motif oriented upward into the core of the binding site, precluding access of a ligand from the ATP binding site into the DFG pocket (Fig 5B). This orientation of the (D)FG was commonly observed in other AF2 DFG-out models, limiting their utility in Type-II ligand discovery when unrefined. Additionally, we noticed a significant difference in the conformation of the activation loop in this model of ABL1 (Fig 5A and 5C), different from the prototypical ‘folded’ conformation often seen in DFG-out structures [28,67]. This model’s ‘extended’ activation loop, more often seen in DFG-in structures [18], obstructed imatinib’s access to the DFG pocket necessary for its proper binding (Fig 5D), another common phenomenon seen in many DFG-out AF2 models we predicted. The removal of this loop could clear this obstruction as a viable option of refining AF2 models for the design of Type-II kinase inhibitors.

In detail, the catalytic domain’s activation loop, in addition to being an important motif to consider in Type-II inhibitor binding, is a significant indicator of the kinase’s activity, where an ‘extended’ conformation is required for the active state and ‘folded’ is considered inactive [15,18]. To study the prevalence of the ‘extended’ activation loop in our AF2 models, we tracked the movement of the activation loop in all confident models by calculating the distance between the HRD-2 and DFG+2 residues, where shorter distances indicate ‘extended’ loops and longer distances mark ‘folding’ of the loop. Indeed, an ‘extended’ conformation of the activation loop was a consistent observation in many of the AF2 models in DFG-out states, where the HRD-2 to DFG+2 distance was similar to that of the DFG-in models, around 7Å (Fig 5E); however, we observed several outliers in our distributions, indicating that many models in the DFG-in conformation still adopted a ‘folded’ activation loop.

## Discussion

While inhibitors targeting the kinases’ ATP binding site may suffer from issues such as promiscuity and low residence time, kinase inhibitors targeting inactive states (e.g., Type-II and Type-I½) can offer several advantages, such as improved pharmacokinetic properties and chemical novelty [20–22,68]. However, the limited number of structures of kinases in inactive states (e.g., DFG-out or ɑC-Helix-out) in the PDB attests to the difficulty of capturing these elusive conformations by experimental methods (Fig 1). Therefore, refined computational prediction methods are necessary to bridge this gap in our structural knowledge for the purpose of advancing drug discovery. Furthermore, a critical evaluation of models predicted for a broad array of kinases from different families is still necessary. Here, we performed the most comprehensive analysis to-date of the kinase conformational space captured by experimentally derived and AI-predicted structures, with the following three key findings:

### The PDB, as well as default AF2 and ESMFold models, exhibit a bias for active kinase conformations

Automated classification of kinase conformations can be achieved with high accuracy [55,69], enabling high-throughput conformational analysis for kinase structure datasets. We determined fractions of kinase conformations in the PDB and AF2 Database, as well as models predicted by ESMFold (Fig 1). Our analysis revealed a significant bias within the PDB for the generally active kinase conformation, CIDI (Fig 1B), which is consistent with previous analyses using different approaches [28,55]. This bias was even more pronounced in the AF2 Database, in agreement with a relatively smaller-scale study [45], and among models predicted by ESMFold (Fig 1C). Furthermore, DFG-out states represented only 10%, 2%, and <1% of the kinases in the PDB, AF2 Database, and ESMFold models, respectively, and together were only a minor fraction of structural data for conformation-specific ligand design. The bias in the models generated by both the AI-based methods likely resulted from the inherent bias in the PDB, which was used for their development and/or evaluation [40,41]. Encouragingly, although the handful of confident AI-generated models in inactive conformations does not fully address the gap in our knowledge of kinase conformations, even a small number of high-quality models in unique conformations may enable the identification of targeted, selective compounds.

### MSA depth influences the diversity of AF2-predicted kinase structures

We explored whether varying the MSA depth, a core parameter of the AlphaFold2 algorithm, could generate more kinase models in inactive states. Our analysis showed that lower MSA depths generally increased conformational diversity and yielded some high-quality models in previously unobserved conformations (Table 1), as shown in similar studies [43,46,70]. For example, NEK9 and CLK3 were predicted with high confidence in the CIDO conformation, and MAP3K4 in the CODI conformation (Fig 2E–2G, respectively), which had not been seen for these proteins in the PDB. This suggests that shallow MSAs fed as input to AF2 may result in fewer structural constraints, thereby enabling the method to sample rare, but possible, conformational states. Further studies should examine what information embedded in sequence alignments drives enhanced sampling of alternative conformations, as our experimentation with AF2 parameters other than its MSA did not result in significant shifts in distributions toward DFG-out states. Furthermore, our efforts did not fully overcome AF2’s bias for predicting kinases in active states, which could be due to the algorithm’s training on the PDB [71]. This may also support the hypothesis that some kinases are less likely to adopt certain conformations, such as DFG-out, as has been suggested elsewhere [72]. It also further supports the notion that newly developed computational tools [49] could potentially address this barrier to explore kinase conformations.

### Select, high-enriching models in unique conformations can be used in rational drug design

A major goal of this study was to generate structural data for the kinome to be used in rational drug design. While several high-scoring models were found in diverse structural conformations, a key question remained: were these models sufficiently accurate for the development of future novel kinase therapeutics? To address this question, we performed an analysis of the quality of the AF2-generated kinase models in different conformations. Interestingly, several models with high TM-Scores [53] (Fig 3A and 3B) that were confident by pLDDT (Fig 3C and 3D) were generated in unique conformations.

In addition, we assessed these models for their utility in rational drug design. Previous smaller scale analyses of the screening ability of AF2 models have been conducted [45,50,51], but our analysis, to our knowledge, concerns the most comprehensive array of kinases to date, revealing previously unknown insights. We anticipated AF2’s training on the PDB, which contains mostly ligand-bound kinase structures, to drive its predictions to emulate more open binding sites that are accessible for ligand binding; however, although on average, the AF2 models enriched only moderately well (avgAUC = 64.58, s.d. = 17.28), a small fraction (5.4% with AUC ≥ 85%) of confident models enriched well enough for rational drug design. This high enrichment occurred more frequently for more confidently predicted models with more open binding sites (Fig 4A and 4B; S2 Table).

Notably, model confidence, as measured by pLDDT, captured the ability of the models to enrich known ligands for confident models (pLDDT ≥ 70) (Fig 4A). This was surprising, because it had been shown previously that methods estimating model quality, such as predicted accuracy of models, do not always correlate with ligand enrichment [73], which is more specifically related to binding site quality [61]. Encouragingly, while a key challenge in analyzing AF2 models is to select the most ‘accurate’ model for further studies, our data suggested that pLDDT can potentially be a useful measure to prioritize models for drug design. Filtering putative models by pLDDT could, therefore, be complementary to other approaches, such as those that use MD simulations [49], statistical potentials, pocket detection algorithms [74–76], local accuracy metrics [77], and graph neural networks [78] to evaluate models for biological relevance.

However, most confident models did not enrich well. Inadequate enrichment could possibly result from inaccurate side chain placement, as has been pointed out before [51]. Similarly, an examination of some models revealed partially occluded binding sites (Figs 4D and 5). Enrichment also depends on possible bias(es) in the ligand dataset used: we clustered known ligands prior to docking to efficiently survey their chemical space, but the binding modes for many inhibitors are unknown, although most are Type-I inhibitors [79]. We posit that AF2 kinase models enrich well if 1) the region between the N- and C-terminal lobes is spacious enough to dock ligands and 2) the active compounds are known to occupy this site. However, our generated AF2 models often do not bear the spatial binding site features necessary to adequately accommodate ligands. Therefore, also considering AF2’s training on PDB data, AF2 models often necessitate further refinement in the same way that many PDB structures often do [51]. For example, the activation loop can be removed if it obstructs the DFG pocket, since it often moves outward in typical DFG-out conformations. Several studies of refined AF2 models for drug discovery have emerged, including the use of implicit experimental density [80], MD simulations [49], and induced-fit docking protocols [81,82] to complement the most confidently predicted models. Thus, the few high-enriching, unobstructed models could represent new starting points for drug design against targets not yet solved in inactive conformations.

Particularly interesting is the fact that AF2 rarely sampled the ‘folded’ activation loop conformation commonly seen in DFG-out PDB structures, which corresponded to the outliers in Fig 5E, and may present an obstacle to kinase DFG-out state modeling. Conversely, many of the outliers in the PDB DFG-in had ‘folded’ activation loops (Fig 5E), such as those of p38 kinase [83] and MAPK14 [84], which was solved without a ligand. Outliers indicating ‘folded’ conformations for this loop were even more abundant in the AF2 DFG-in models generated at various MSA depths. These observations may suggest that activation loop movement is not directly tied to a change in the DFG conformation, which is also supported by the observation of a broad range of distances in DFG-out PDB structures (Fig 5E). Further analyses of factors dictating movements of both motifs, some of which have recently emerged [15,67], are warranted, considering the importance of both for determining a kinase’s activity.

Taken together, our results suggest that a rigorous and intentional interrogation of AI-predicted models by a trained expert remains an important step in kinase drug design. Specifically, while AI-based methods can make the structural characterization of kinases more efficient, the translation of these models into drug discovery campaigns still likely requires expert manual inspection to verify the placement of binding site sidechains, openness of druggable pockets, and large conformational changes [51]. Furthermore, AI-based methods do not negate the utility of experimentally determined structures; the tools that generated our models can supplement available experimental data in an attempt to bridge the vast, untraversed gap between kinase structural biology and the kinome’s unexplored conformational space. The recently published AlphaFold3 (AF3) [85] is a promising tool for conformation-specific drug design, which has the ability to predict structures of protein-ligand complexes. However, AF3 must be subject to rigorous critique and validation. Upon DeepMind’s release of AF3’s standalone version, an obvious direction for research would be studying the conformational diversity of AF3 models at different MSA depths. It would also be interesting to discover how enrichment using AF3-predicted kinase models compares to the results presented here. Finally, it would be worth studying how the prediction of inactivating kinase ligands influences movement of the activation loop. Nevertheless, our work here provides the most comprehensive view of AF2’s impact and utility in the computational design of kinase therapeutics. Also, as more kinase structures are experimentally solved in alternative conformations, and novel ligands targeting these states are developed, newly trained machine learning (ML) algorithms will likely be more generalizable, further driving drug discovery.

## Materials and methods

### Prediction using AlphaFold2

High-throughput AlphaFold2 model generation was performed using the Minerva high-performance computing facilities at the Icahn School of Medicine at Mount Sinai. An NVIDIAA100_PCIE_40GB GPU was used to run the method. Input sequences were derived from a previously published multiple sequence alignment (MSA) of 497 kinase domains [86]. Ensembl BioMart [87], a web-based tool allowing the extraction of various biological data, was employed to match the kinase gene names to their associated Ensembl protein IDs. Furthermore, using BioMart, the PDB IDs of protein structures pertaining to the aggregated kinase protein IDs were retrieved on March 15^th^, 2023, and the structural models matching these IDs were downloaded via the PDB’s bulk download tool in the CIF format.

We predicted AlphaFold2 models of kinase domains at a range of MSA depths. These models were generated using a modified implementation of the AlphaFold2_advanced ColabFold repository [57]. The specific modifications we made were that the ‘max_recycles’ and ‘is_training’ variables were set to 1 to reduce structural refinement and ‘True’ to enable model dropout, respectively, for most predictions. For modeling at each MSA depth, the max_msa variable was set to “{desired MSA depth}:{double desired MSA Depth}.” The maximum number of models output per kinase at each MSA depth was set to five. The resultant models were ranked by the pLDDT score calculated by AF2. Additional runs of AlphaFold2 varied the number of random_seeds to generate more models per MSA depth, as done by da Silva et al [46]. To enable further study of and rational drug design efforts against such targets, all AF2-generated models analyzed here have been made available at https://kinametrix.com/ under the ‘AlphaFold2 Kinase Structures’ tab with processing scripts accessible through our GitHub repository (https://github.com/schlessinger-lab/af2_kinase_conformations/).

Memory limitations forbade successful predictions of models of MASTL at a depth of 512. All predicted models were visualized using the PyMOL Molecular Graphics System (version 2.5.4) hosted by Schrödinger, LLC. Electrostatic maps were generating using the Adaptive Poisson-Boltzmann Solver (APBS) plugin [88].

### ESMFold model generation

Models were predicted based on sequences from the same multiple sequence alignment of 497 kinase domains [86] using the v1 implementation of ESMFold [41] with default parameters.

### Kinase conformation classification

Initial conformational classification of PDB structures and AF2 and ESMFold models of kinases were conducted using *Kincore* [55] and *Kinformation* [69]. Due to its improved coverage of the kinome, *Kincore*’s results were shown and analyzed throughout this paper. This method classifies kinases into eight conformations based on the DFG motif and ɑC-Helix movements, which we grouped into the following six conformations to simplify classification by DFG-in and parse by ɑC-Helix conformation: DFG-in/ɑC-Helix-in (CIDI), DFG-in/ɑC-Helix-out (CODI) (DFG-in clusters in *Kincore*: BLAminus, BLAplus, ABAminus, BLBminus, BLBplus, and BLBtrans), DFG-out/ɑC-Helix-in (CIDO), DFG-out/ɑC-Helix-out (CODO) (DFG-out cluster: BBAminus in *Kincore*), DFGinter and Unassigned [15]. Kincore could not identify the residues of and near the DFG motif (or substituted residues) used for conformation classification for a total of twelve kinases, so, these were excluded from the analyses of conformations. A total of 5,136 structures and 494 models were classified from the PDB and AF2 Database, respectively. Certain UniProt IDs were associated with multiple kinase models downloaded from the AF2 Database, rarely in more than one conformation; to calculate the fractional contribution to each conformation by each kinase containing these domains, 1,000 random samples of one conformation per kinase domain were taken from all conformations for each ID. These fractional contributions were calculated 10 times and averaged to calculate an overall contribution that was added to the corresponding conformation fractions in distribution calculations. In total, 2,425 AlphaFold2-predicted models were classified for each MSA depth, except for the depth of 512, for which 2,420 models were classified (due to memory limitations, AF2 failed to generate models for MASTL). A total of 486 ESMFold models were classified into the six conformations. Fractions of generated models by conformation were calculated as percents by dividing the sum of models of each conformation type by the sum of all classified models at each depth. Mapping of conformation counts to kinases and their groups was performed using KinMap [58]. Comparison of kinase conformations represented in the PDB and at an MSA depth of 8, displayed in these maps, was performed via a one-sided, paired t-test (p-value (Average_AF2_ > Average_PDB_)). All conformations predicted for each kinase with AF2 are available in S5 Table by MSA.

### Dihedral plots

Plots of pseudo-dihedral angles used to visualize movement of the DFG motif were generated using Möbitz [89]. Dihedral angles were calculated using the Cɑ carbons of 4 consecutive residues near or including the DFG motif. Biopython 1.81 [90] was used to calculate the coordinates of the required Cɑ carbons. The DFG Asp through the first residue after the DFG motif was used to define the dihedral on the X-axes of S6A–S6C Fig, and the two residues preceding the DFG motif through the DFG Phe were used to define the same on the corresponding Y-axes.

### Statistical analyses

Comparisons of fractions of specific conformations were performed by bootstrapping from conformational distributions of, and counting the number of the conformation sampled, all 1,000 times. P-values of these comparisons were calculated using one-sided Wilcoxon rank-sum tests. The Chi-squared, Wilcoxon rank-sum, Fisher’s exact and paired t-tests used in our analyses were performed using the ‘chisq.test,’ ‘wilcox.test,’ ‘fisher.test,’ and ‘t.test,’ functions, respectively, from R 4.9.0. The specific analyses performed between different datasets are described in S1 Fig. One-sample t-tests were performed using the ‘ttest_1samp’ function from Scikit-learn [91].

### TM-Score calculations

TM-Score is a metric used to compare structural similarity and is sensitive to small, local variations affecting the score of the global fold [46]. In our study, TM-Scoring of AF2 models against PDB structures was performed using the ‘tmtools’ module and a series of helper functions [92]. Since the sequences we used to model kinases rarely contained insertions and the length of the reference model is used to weight the final score, we used the AF2 models as the reference model. This was done to more accurately assess the similarities between AF2-predicted and PDB kinase domains by excluding flexible insertions in PDB structures from comparisons. To focus on kinases with catalytic activity and find catalytic PDB subunits, TM-Score comparisons were only conducted between structures sharing a specific ‘DFG’ motif. Distributions of TM-Scores were calculated as averages of the highest TM-Scores for AF2 models compared to PDB structures of the same kinases for each MSA depth and structural conformation.

### Kinase model enrichment analyses

All confident models of a set of 23 kinases taken from the Directory of Useful Decoys–Enhanced (DUD-E) benchmark study [62] underwent enrichment studies using known ligands taken from the ChEMBL database [63]. Curation of known ligands for each kinase by IC_50_, K_I_, or K_D_ measurements and nanomolar potency was accomplished using a publicly available Jupyter notebook [93]. The filtered ligands for each kinase underwent k-means clustering using a publicly available script [94] into 50 clusters, the centers of which were used to generate 50 decoys each using the DUD-E webserver [62]. The AF2-predicted models and combined cluster centers’ and decoys’ SMILES codes underwent protein and ligand preparation, respectively, before being docked using Glide [64], all using the Schrödinger suite. Our ligand preparation included sampling varied ionization states using default parameters of the Epik [95] utility at physiological pH ± 2 units. For all proteins undergoing enrichment, models of each kinase were aligned to the model with the highest pLDDT for that kinase. A centroid for docking was then defined using a centroid between three residues in the center of the cleft between the N- and C-terminal lobes. Following docking, enrichment figures were generated by plotting the percent of known ligands identified, versus the top percent of the ranked database examined, considering only the top poses of all ligands. Calculated AUCs were multiplied by 100 to fit within a 0 to 100 percent range. Box plots summarizing the enrichment results by pLDDT and conformation were generated using Matplotlib [96].

### Calculation of kinase model activation loop movement

For all PDB structures confident AF2 models at all MSA depths in DFG-in and DFG-out conformations, we estimated the extent of each model’s activation loop movement by calculating the distance between the Cɑ carbons of the HRD–2 and DFG+2 residues, as has been done previously [67]. The location of the DFG motif was determined using Kincore’s alignment and classification algorithm [55]. Coordinates for the Cɑ atoms were determined using Biopython [90].

## Supporting information

S1 Fig Workflow diagram for analysis of kinase structural models. Kinase structures are downloaded from the Protein Data Bank (PDB) before being classified together and by their respective groups. The same is performed on kinase models downloaded from the AlphaFold2 Protein Structure Database (‘AF2 Database’) and generated through ColabFold and ESMFold. Distributions of structures and/or models are then compared via various statistical tests.
(DOCX)

S2 Fig Distribution of human kinase models predicted by ESMFold by conformation (n = 486). Models generated by ESMFold and classified to a conformation by Kincore [55], as described in Methods. showed a strong preference for the active state (CIDI), significantly more so than the PDB (p-value PDB_CIDI_ < ESMFold_CIDI_ < 2.2 x 10^−16^ or AlphaFold2 (p-value AF2_CIDI_ < ESMFold_CIDI_ < 2.2 x 10^−16^), and similarly low preference for DFG-out states (CIDO and CODO), even lower than the PDB (p-value PDB_DFG-out_ > ESMFold_DFG-out_ < 2.2 x 10^−16^) or AlphaFold2 (p-value AF2_DFG-out_ > ESMFold_DFG-out_ = 5.47 x 10^−281^). P-values were obtained by a one-sided Wilcoxon rank-sum test.
(DOCX)

S3 Fig Distributions of conformations of only A) AF2 Database and B) ESMFold models of human kinases with deposited experimental structures in the PDB. The AF2 and ESMFold distributions were compared via a Fisher exact test. The resultant p-values of 0.751 and 0.913 for the AF2 Database and ESMFold overlap indicated that these distributions are not statistically different from all kinase models taken from the whole AF2 Database and ESMFold dataset, respectively.
(DOCX)

S4 Fig Fractional distributions of kinase conformations predicted by ESMFold by group. Kinases are grouped into the following groups: AGC (PKA, PKG, PKC families;), CAMK (Calcium/calmodulin-dependent), CK1 (Casein kinase 1), CMGC (CDK, MAPK, GSK3, CLK families), STE (Sterile 7, Sterile 11, Sterile 20 kinases), TK (Tyrosine kinase; Tyrosine kinase-like), and Other. As in the PDB (Fig 1D) and AF2 Databases (Fig 1E), the active (CIDI) conformations were the most abundant across all families, while DFG-out conformations (i.e., CIDO, CODO) were substantially under-represented.
(DOCX)

S5 Fig Distributions of conformations of AF2 models generated using a custom MSA. A custom MSA was constructed from sequences of kinases predicted in at least one DFG-out conformation using AF2 at an MSA depth of 8. The counts of sequences in the custom MSA were gradually reduced to create new MSAs of varying depths: 161, 32, 16, 8, and 4. Fisher’s exact tests were used to compare each distribution to that of the AF2 Database, and all the resultant p-values indicated that none of these distributions were significantly different from that of the latter.
(DOCX)

S6 Fig Distribution of AF2-predicted models in terms of the movement of the DFG motif, as defined by pseudo-dihedral angles, for AF2-predicted models. For models predicted at MSA depths of (A) 8, (B) 512, and (C) 2, pseudo-dihedral angles are defined by Möbitz [89]. The X-axis is defined by the pseudo-dihedral angle constructed by the Cɑ carbons of the DFG-Asp, DFG-Phe, DFG-Gly and DFG+1 residues, while the Y-axis is defined by the angles of the DFG-2, DFG-1, DFG-Asp and DFG-Phe residues. (A) Distribution of all models predicted at an MSA depth of 8 with pLDDT > 70, categorized by their adopted conformation. (B) As defined in A, dihedral angles plot of all AF2 models with pLDDT > 70 predicted at an MSA depth of 512. (C) Dihedral angles plot of all AF2 models predicted at an MSA depth of 2 with pLDDT > 70. (D) Examples of confident ‘Unassigned’ models are illustrated, where red and blue atoms indicate oxygen and nitrogen atoms, respectively.
(DOCX)

S1 Text Analysis of AlphaFold2 (AF2) protein kinase models by flexibility of their DFG motifs. Results and methodology regarding our analysis of the DFG motif movement in AF2 models at MSA depths 8 (S6A Fig), 512 (S6B Fig), and 2 (S6C Fig) are summarized. Examples of confident Unassigned models of CAMK2A, DDR2, and ULK3 are described (S6D Fig).
(DOCX)

S1 Table Comparison of distributions of models predicted by AF2 at various MSA depths to that of the AF2 Database. P-values were obtained using the Fisher’s exact test. ^a^ MSA Depth corresponds to the number of sequences included in the multiple sequence alignment (MSA) used as input for AF2 through ColabFold. ^b^ At a significance level of 0.05, all p-values indicate that only the distribution at an MSA depth of 512 was statistically similar to that of the AF2 Database. All others were significantly different.
(DOCX)

S2 Table Comparison of conformational fraction distributions of kinase models generated by AF2 at different MSA depths. P-values were calculated using the Chi-squared test. At a Bonferroni-corrected significance level of p < 0.00238, all distributions by each MSA depth are statistically different from each other. ^**a**^ MSA Depth corresponds to the number of sequences included in the Multiple Sequence Alignment (MSA) used as input for AF2 through ColabFold.
(DOCX)

S3 Table Comparison of conformation fractions of kinase models deposited in AF2 Database and predicted by AF2 at MSA depth 8. P-values were calculated using the one-sided Wilcoxon rank-sum test at a significance level of 0.05. ^a^ Group indicates kinase group within which comparisons are being made. ^b^ ‘CIDI’ indicates the significance of the p(AF2_CIDI_ > 8MSA_CIDI_) comparison of ‘CIDI’ fractions. All other comparisons indicate comparison by p(AF2_CONF_ < 8MSA_CONF_).
(DOCX)

S4 Table Average and max AUCs for enrichment of AF2 kinase models. ^**a**^ Name of kinase in enrichment analysis. ^**b**^ Average area-under-the-curve (AUC) from enrichment plots of all of each kinase’s AF2 models. ^**c**^ Standard deviation of the distribution of enrichment AUCs for each kinase’s AF2 models. ^**d**^ Highest AUC of all enrichment plots for each kinase’s AF2 models.
(DOCX)

S5 Table Predicted conformations by AF2 for protein kinases at various MSA depths. ^a^ MSA depths are separated by tabs. ^b^ Kinase indicates the specific protein kinase being analyzed. ^c^ Group indicates to the kinase group to which the specific protein kinase belongs. ^d^ Conformation(s) indicates all predicted conformations for the specific protein kinase at the MSA depth.
(XLSX)
