# Supplementary material for: A comprehensive exploration of the druggable conformational space of protein kinases using AI-predicted structures
Source: PLoS Comput Biol. 2024 Jul 24;20(7):e1012302. doi: 10.1371/journal.pcbi.1012302 (PMC11268620; doi:10.1371/journal.pcbi.1012302)
Supplement: S1 Fig — Kinase structures are downloaded from the Protein Data Bank (PDB) before being classified together and by their respective groups. The same is performed on kinase models downloaded from the AlphaFold2 Protein Structure Database (‘AF2 Database’) and generated through ColabFold and ESMFold. Distributions of structures and/or models are then compared via various statistical tests. (DOCX) [file pcbi.1012302.s001.docx]

**
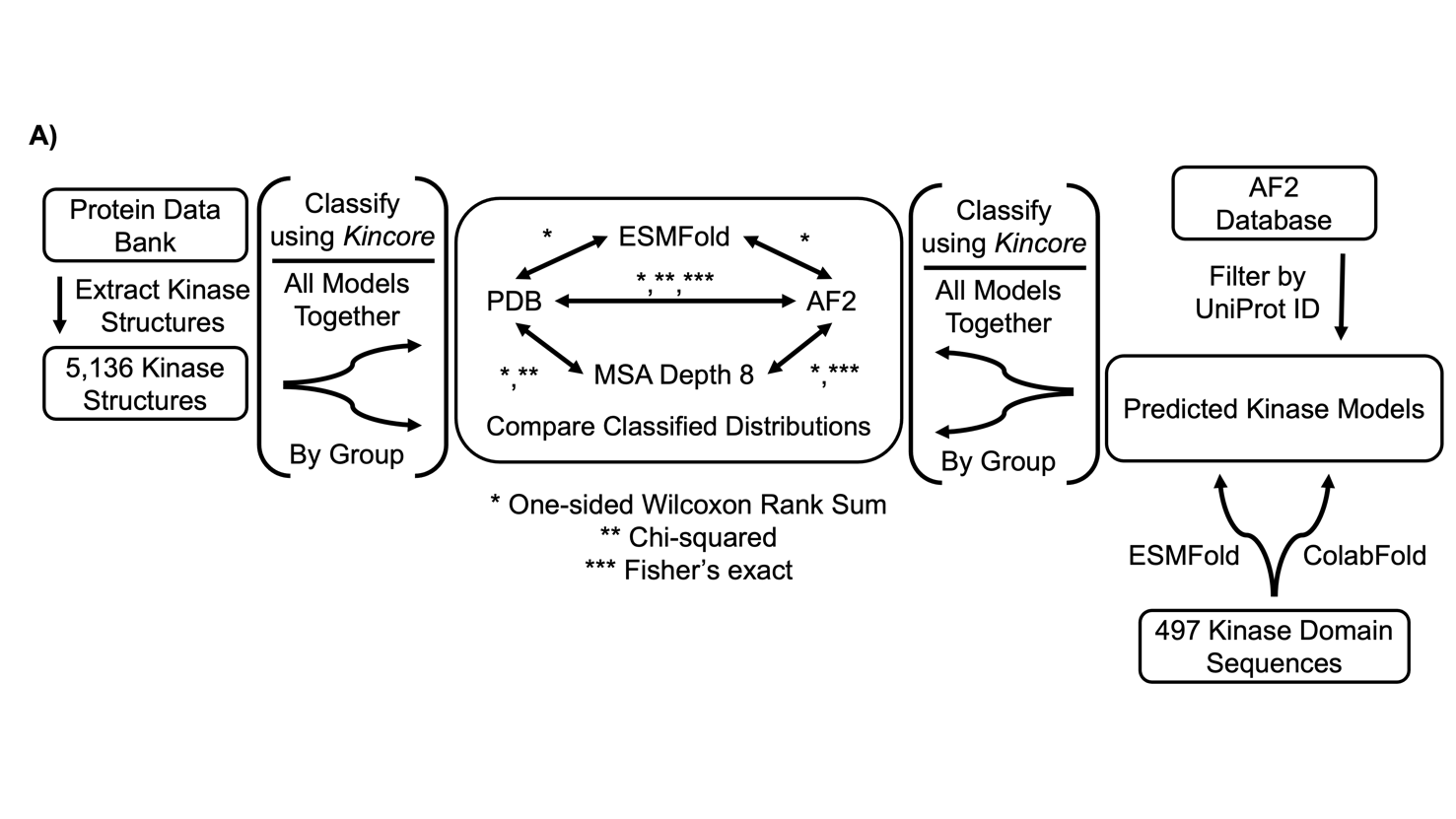
**

**S1 Fig. Workflow diagram for analysis of kinase structural models.**

Kinase structures are downloaded from the Protein Data Bank (PDB) before being classified together and by their respective groups. The same is performed on kinase models downloaded from the AlphaFold2 Protein Structure Database (‘AF2 Database’) and generated through ColabFold and ESMFold. Distributions of structures and/or models are then compared via various statistical tests.
