# Supplementary material for: A comprehensive exploration of the druggable conformational space of protein kinases using AI-predicted structures
Source: PLoS Comput Biol. 2024 Jul 24;20(7):e1012302. doi: 10.1371/journal.pcbi.1012302 (PMC11268620; doi:10.1371/journal.pcbi.1012302)
Supplement: S2 Fig — Models generated by ESMFold and classified to a conformation by Kincore [55], as described in Methods. showed a strong preference for the active state (CIDI), significantly more so than the PDB (p-value PDBCIDI < ESMFoldCIDI < 2.2 x 10^−16^ or AlphaFold2 (p-value AF2CIDI < ESMFoldCIDI < 2.2 x 10^−16^), and similarly low preference for DFG-out states (CIDO and CODO), even lower than the PDB (p-value PDBDFG-out > ESMFoldDFG-out < 2.2 x 10^−16^) or AlphaFold2 (p-value AF2DFG-out > ESMFoldDFG-out = 5.47 x 10−281). P-values were obtained by a one-sided Wilcoxon rank-sum test. (DOCX) [file pcbi.1012302.s002.docx]

**
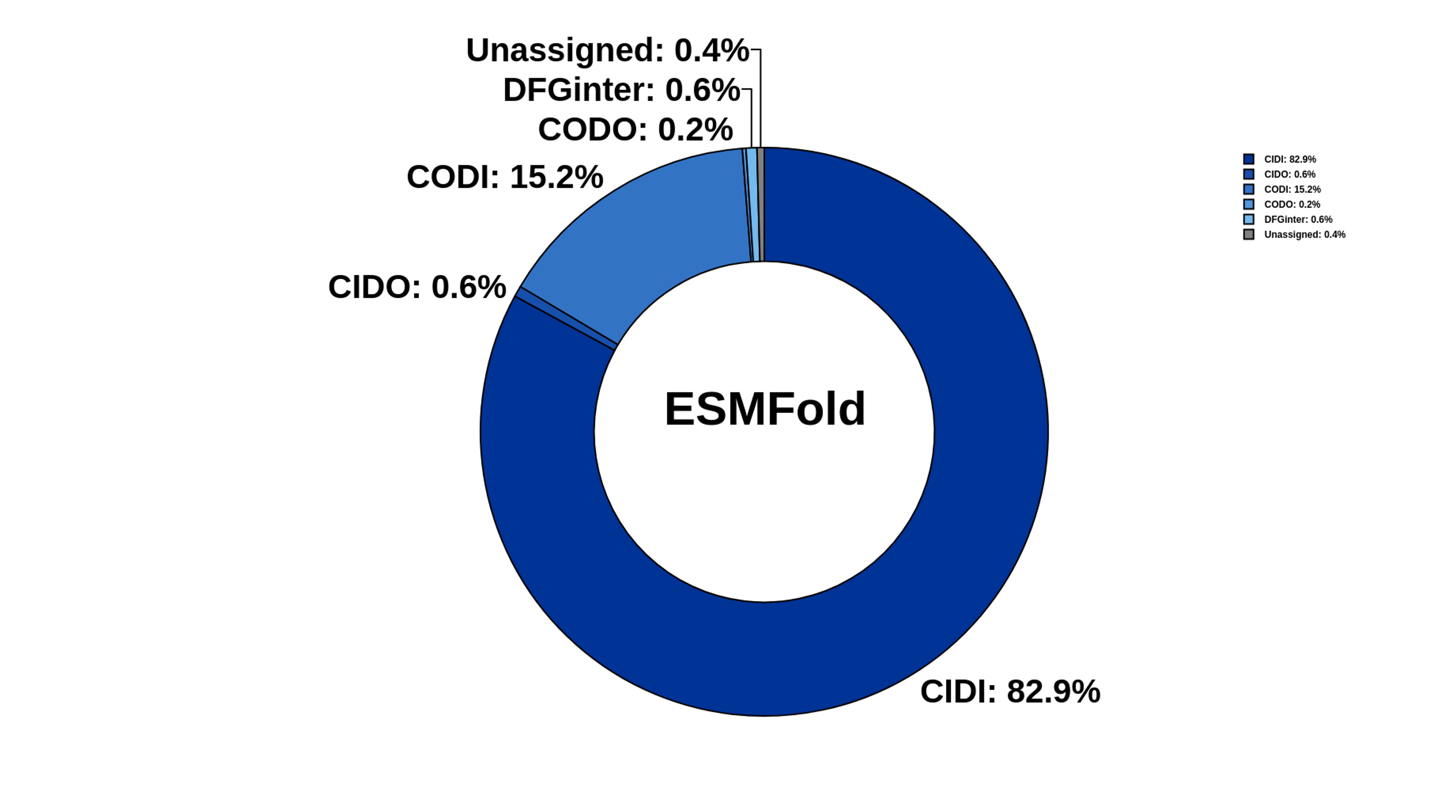
**

**S2 Fig. Distribution of human kinase models predicted by ESMFold by conformation (n = 486)**.

Models generated by ESMFold and classified to a conformation by Kincore (55), as described in Methods. These models showed a strong preference for the active state (CIDI), significantly more so than the PDB (p-value PDB_CIDI_ < ESMFold_CIDI_ < 2.2 x 10^-16^) and AlphaFold2 (p-value AF2_CIDI_ < ESMFold_CIDI_ < 2.2 x 10^-16^), and a low preference for DFG-out states (CIDO and CODO), even lower than the PDB (p-value PDB_DFG-out_ > ESMFold_DFG-out_ < 2.2 x 10^-16^) and AlphaFold2 (p-value AF2_DFG-out_ > ESMFold_DFG-out_ = 5.47 x 10^-281^). P-values were calculated using a one-sided Wilcoxon rank-sum test.
