# Supplementary material for: A comprehensive exploration of the druggable conformational space of protein kinases using AI-predicted structures
Source: PLoS Comput Biol. 2024 Jul 24;20(7):e1012302. doi: 10.1371/journal.pcbi.1012302 (PMC11268620; doi:10.1371/journal.pcbi.1012302)
Supplement: S3 Fig — The AF2 and ESMFold distributions were compared via a Fisher exact test. The resultant p-values of 0.751 and 0.913 for the AF2 Database and ESMFold overlap indicated that these distributions are not statistically different from all kinase models taken from the whole AF2 Database and ESMFold dataset, respectively. (DOCX) [file pcbi.1012302.s003.docx]

**
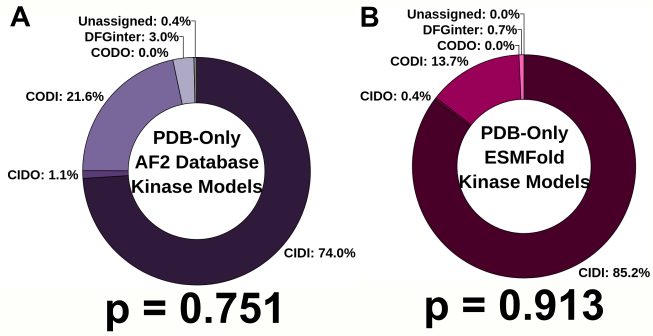
**

**S3 Fig. Distributions of conformations of only A) AF2 Database and B) ESMFold models of human kinases with deposited experimental structures in the PDB.**

The AF2 and ESMFold distributions were compared via a Fisher exact test. The resultant p-values of 0.751 and 0.913 for the AF2 Database and ESMFold overlap indicated that these distributions are not statistically different from all kinase models taken from the whole AF2 Database and ESMFold dataset, respectively.
