# Supplementary material for: A comprehensive exploration of the druggable conformational space of protein kinases using AI-predicted structures
Source: PLoS Comput Biol. 2024 Jul 24;20(7):e1012302. doi: 10.1371/journal.pcbi.1012302 (PMC11268620; doi:10.1371/journal.pcbi.1012302)
Supplement: S4 Fig — Kinases are grouped into the following groups: AGC (PKA, PKG, PKC families;), CAMK (Calcium/calmodulin-dependent), CK1 (Casein kinase 1), CMGC (CDK, MAPK, GSK3, CLK families), STE (Sterile 7, Sterile 11, Sterile 20 kinases), TK (Tyrosine kinase; Tyrosine kinase-like), and Other. As in the PDB (Fig 1D) and AF2 Databases (Fig 1E), the active (CIDI) conformations were the most abundant across all families, while DFG-out conformations (i.e., CIDO, CODO) were substantially under-represented. (DOCX) [file pcbi.1012302.s004.docx]

**
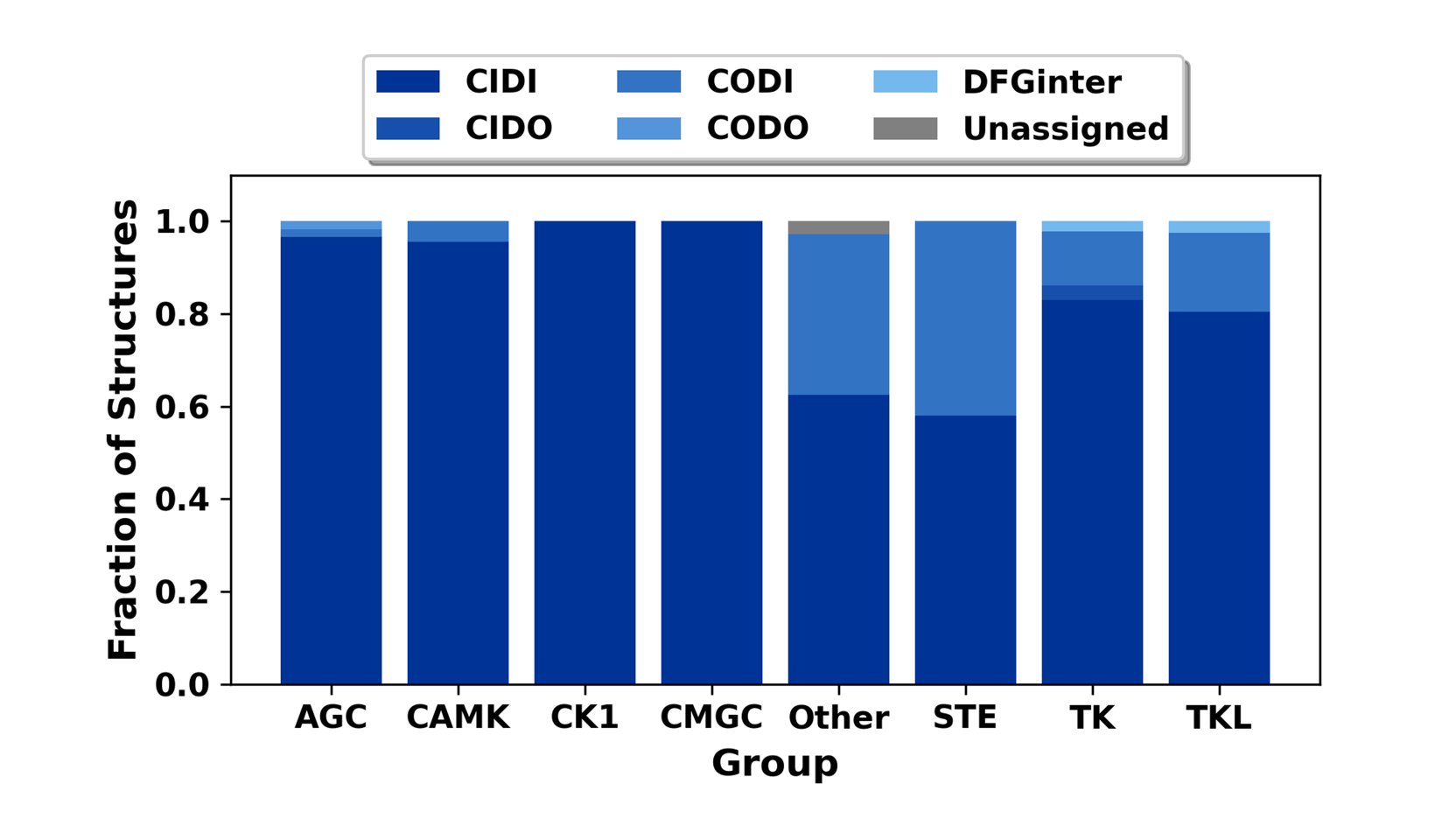
**

**S4 Fig. Fractional distributions of kinase conformations predicted by ESMFold** **by group.**

Kinases were grouped into the following groups: AGC (PKA, PKG, PKC families;), CAMK (Calcium/calmodulin-dependent), CK1 (Casein kinase 1), CMGC (CDK, MAPK, GSK3, CLK families), STE (Sterile 7, Sterile 11, Sterile 20 kinases), TK (Tyrosine kinase; Tyrosine kinase-like), and Other. As in the PDB (Fig 1D) and AF2 Databases (Fig 1E), the active (CIDI) conformations were the most abundant across all families, while DFG-out conformations (i.e., CIDO and CODO) were substantially under-represented.
