# Supplementary material for: A comprehensive exploration of the druggable conformational space of protein kinases using AI-predicted structures
Source: PLoS Comput Biol. 2024 Jul 24;20(7):e1012302. doi: 10.1371/journal.pcbi.1012302 (PMC11268620; doi:10.1371/journal.pcbi.1012302)
Supplement: S5 Fig — A custom MSA was constructed from sequences of kinases predicted in at least one DFG-out conformation using AF2 at an MSA depth of 8. The counts of sequences in the custom MSA were gradually reduced to create new MSAs of varying depths: 161, 32, 16, 8, and 4. Fisher’s exact tests were used to compare each distribution to that of the AF2 Database, and all the resultant p-values indicated that none of these distributions were significantly different from that of the latter. (DOCX) [file pcbi.1012302.s005.docx]

**
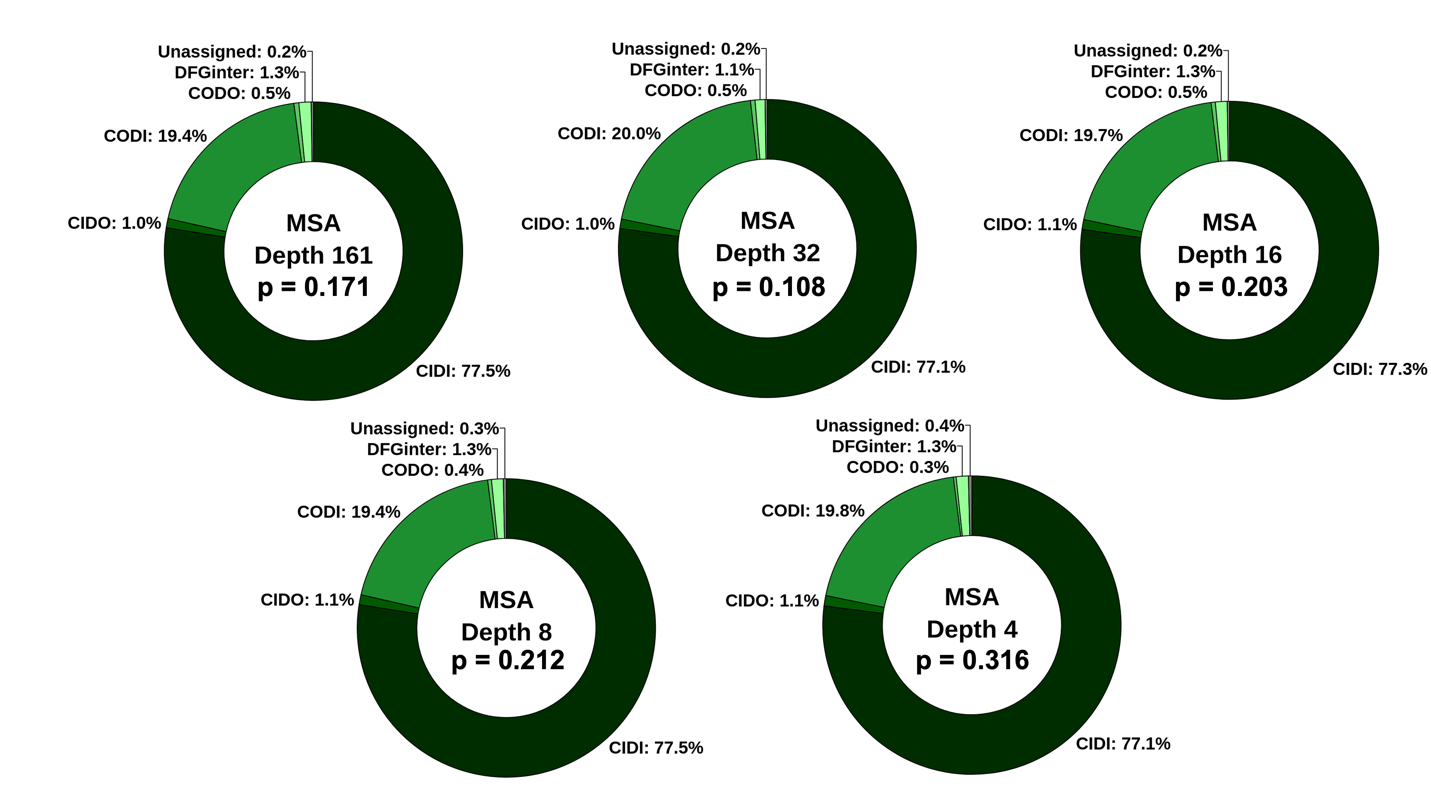
S5 Fig.** **Distributions of conformations of AF2 models generated using a custom MSA.**

A custom MSA was constructed from sequences of kinases predicted in at least one DFG-out conformation using AF2 at an MSA depth of 8. The counts of sequences in the custom MSA were gradually reduced to create new MSAs of varying depths: 161, 32, 16, 8, and 4. Fisher’s exact tests were used to compare each distribution to that of the AF2 Database, and all the resultant p-values indicated that none of these distributions were significantly different from that of the latter.
