# Supplementary material for: A comprehensive exploration of the druggable conformational space of protein kinases using AI-predicted structures
Source: PLoS Comput Biol. 2024 Jul 24;20(7):e1012302. doi: 10.1371/journal.pcbi.1012302 (PMC11268620; doi:10.1371/journal.pcbi.1012302)
Supplement: S6 Fig — For models predicted at MSA depths of (A) 8, (B) 512, and (C) 2, pseudo-dihedral angles are defined by Möbitz [89]. The X-axis is defined by the pseudo-dihedral angle constructed by the Cɑ carbons of the DFG-Asp, DFG-Phe, DFG-Gly and DFG+1 residues, while the Y-axis is defined by the angles of the DFG-2, DFG-1, DFG-Asp and DFG-Phe residues. (A) Distribution of all models predicted at an MSA depth of 8 with pLDDT > 70, categorized by their adopted conformation. (B) As defined in A, dihedral angles plot of all AF2 models with pLDDT > 70 predicted at an MSA depth of 512. (C) Dihedral angles plot of all AF2 models predicted at an MSA depth of 2 with pLDDT > 70. (D) Examples of confident ‘Unassigned’ models are illustrated, where red and blue atoms indicate oxygen and nitrogen atoms, respectively. (DOCX) [file pcbi.1012302.s006.docx]

**
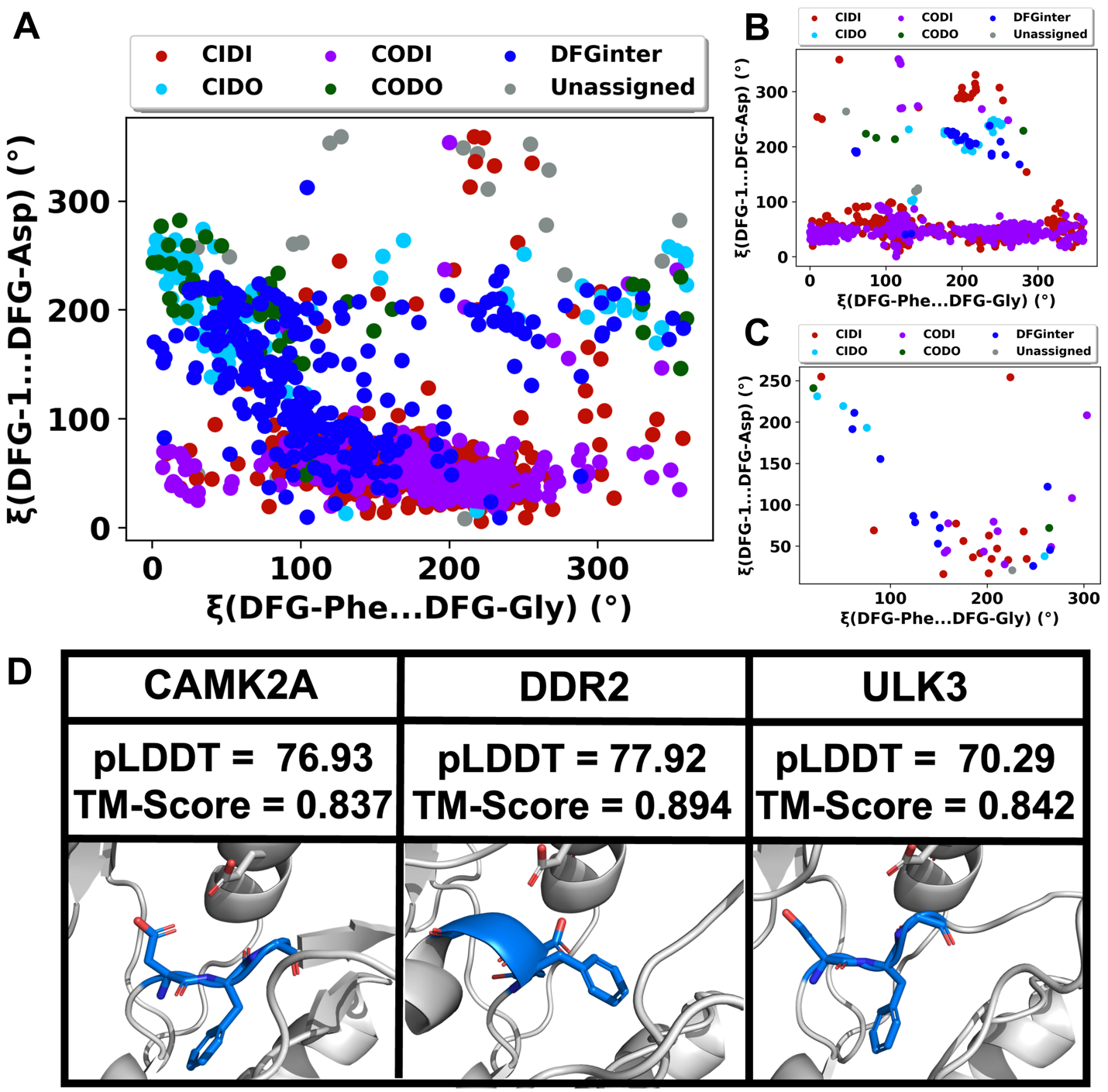
**

**S5 Fig.** **Distribution of AF2-predicted models in terms of the movement of the DFG motif, as defined by pseudo-dihedral angles, for AF2-predicted models.**

For models predicted at MSA depths of (A) 8, (B) 512, and (C) 2, pseudo-dihedral angles are defined by Möbitz (89). The X-axis is defined by the pseudo-dihedral angle constructed by the Cɑ carbons of the DFG-Asp, DFG-Phe, DFG-Gly and DFG+1 residues, while the Y-axis is defined by the angles of the DFG-2, DFG-1, DFG-Asp and DFG-Phe residues. (A) Distribution of all models predicted at an MSA depth of 8 with pLDDT > 70, categorized by their adopted conformation. (B) As defined in A, dihedral angles plot of all AF2 models with pLDDT > 70 predicted at an MSA depth of 512. (C) Dihedral angles plot of all AF2 models predicted at an MSA depth of 2 with pLDDT > 70. (D) Examples of confident ‘Unassigned’ models are illustrated, where red and blue atoms indicate oxygen and nitrogen atoms, respectively.
