# Supplementary material for: A comprehensive exploration of the druggable conformational space of protein kinases using AI-predicted structures
Source: PLoS Comput Biol. 2024 Jul 24;20(7):e1012302. doi: 10.1371/journal.pcbi.1012302 (PMC11268620; doi:10.1371/journal.pcbi.1012302)
Supplement: S1 Text — Results and methodology regarding our analysis of the DFG motif movement in AF2 models at MSA depths 8 (S6A Fig), 512 (S6B Fig), and 2 (S6C Fig) are summarized. Examples of confident Unassigned models of CAMK2A, DDR2, and ULK3 are described (S6D Fig). (DOCX) [file pcbi.1012302.s007.docx]

**S1 Text. Analysis of AlphaFold2 (AF2) protein kinase models by flexibility of their DFG motifs.**

Results and methodology regarding our analysis of the DFG motif movement in AF2 models at MSA depths 8 (Fig A in S6 Fig), 512 (Fig B in S6 Fig), and 2 (Fig C in S6 Fig) are summarized. Examples of confident Unassigned models of CAMK2A, DDR2, and ULK3 are described (Fig D in S6 Fig).

In addition to model quality, it was also important to assess how well AF2 explored the conformational flexibility of the movement of the DFG motif. This movement determines the biophysical features (e.g., shape and volume) of the ATP binding site, which are relevant for the development of particular kinase inhibitor types (e.g., Type-I and -I1⁄2) (26, 27). For this, we analyzed the reliable models predicted (i.e., pLDDT > 70) by AF2 at an MSA depth of 8 (Fig A in S6 Fig) in terms of pseudo-dihedral angles involving the DFG backbone (89). Briefly, these pseudo-dihedral angles capture the movement of the DFG motif and distinguish between DFG-in and DFG-out models. In Fig A of S6 Fig, the DFG-in conformations (red and purple) clustered toward the bottom of the plots, as expected, and DFG-out models (cyan and green) clustered in a line near the middle in the same way. DFGinter models (blue) spanned the conformational space between the two states.

Notably, for the MSA depth of 512, we observed a limited exploration of DFG movement in the DFG-out and Unassigned conformational space (Fig B in S6 Fig), indicated by narrower clusters of models in each region and fewer DFG-out models. In contrast, the MSA depth of 2 showed wider exploration of the DFG motif’s conformational space, but it is difficult to make definitive conclusions, as far fewer models were confidently predicted at this depth (Fig C in S6 Fig). These observations demonstrated that reliable AF2- predicted models captured the known conformational space of the DFG motif but also some diverse and potentially druggable conformations (i.e., via the ATP binding site) outside of standard groupings. Unassigned models were generated ubiquitously throughout the DFG conformational space (Fig A in S6 Fig), including confident models of CAMK2A, DDR2, and ULK3 at an MSA depth of 8 (Fig D in S6 Fig). These models of these three kinases illustrate that AF2’s predictions explore other diverse conformations.

**References**

1. Seeliger MA, Nagar B, Frank F, Cao X, Henderson MN, Kuriyan J. c-Src binds to the cancer drug imatinib with an inactive Abl/c-Kit conformation and a distributed thermodynamic penalty. Structure. 2007;15(3):299-311.

2. Seeliger MA, Ranjitkar P, Kasap C, Shan Y, Shaw DE, Shah NP, et al. Equally potent inhibition of c-Src and Abl by compounds that recognize inactive kinase conformations. Cancer Res. 2009;69(6):2384-92.

3. Wood ER, Truesdale AT, McDonald OB, Yuan D, Hassell A, Dickerson SH, et al. A unique structure for epidermal growth factor receptor bound to GW572016 (Lapatinib): relationships among protein conformation, inhibitor off-rate, and receptor activity in tumor cells. Cancer Res. 2004;64(18):6652-9.

4. Möbitz H. The ABC of protein kinase conformations. Biochim Biophys Acta. 2015;1854(10 Pt B):1555-66.
