# Supplementary material for: A comprehensive exploration of the druggable conformational space of protein kinases using AI-predicted structures
Source: PLoS Comput Biol. 2024 Jul 24;20(7):e1012302. doi: 10.1371/journal.pcbi.1012302 (PMC11268620; doi:10.1371/journal.pcbi.1012302)
Supplement: S1 Table — P-values were obtained using the Fisher’s exact test. a MSA Depth corresponds to the number of sequences included in the multiple sequence alignment (MSA) used as input for AF2 through ColabFold. b At a significance level of 0.05, all p-values indicate that only the distribution at an MSA depth of 512 was statistically similar to that of the AF2 Database. All others were significantly different. (DOCX) [file pcbi.1012302.s008.docx]

**S1 Table. Comparison of distributions of models predicted by AF2 at various MSA depths to that of the AF2 Database.**

| **MSA Depth^a^** | **p-value^b^** |
| --- | --- |
| **512** | **0.499** |
| **128** | **0.0045** |
| **32** | **0.0045** |
| **16** | **0.0045** |
| **8** | **0.0045** |
| **4** | **0.0045** |
| **2** | **0.0045** |

P-values were obtained using the Fisher’s exact test.

^a^ MSA Depth corresponds to the number of sequences included in the multiple sequence alignment (MSA) used as input to AF2 through ColabFold.

^b^ At a significance level of 0.05, all p-values indicate that only the distribution at an MSA depth of 512 was statistically similar to that of the AF2 Database. All others were significantly different.
