# Supplementary material for: A comprehensive exploration of the druggable conformational space of protein kinases using AI-predicted structures
Source: PLoS Comput Biol. 2024 Jul 24;20(7):e1012302. doi: 10.1371/journal.pcbi.1012302 (PMC11268620; doi:10.1371/journal.pcbi.1012302)
Supplement: S2 Table — P-values were calculated using the Chi-squared test. At a Bonferroni-corrected significance level of p < 0.00238, all distributions by each MSA depth are statistically different from each other. a MSA Depth corresponds to the number of sequences included in the Multiple Sequence Alignment (MSA) used as input for AF2 through ColabFold. (DOCX) [file pcbi.1012302.s009.docx]

**S2 Table. Comparison of conformational fraction distributions of kinase models generated by AF2 at different MSA depths.**

| **MSA Depth^a^** | **512** | **128** | **32** | **16** | **8** | **4** | **2** |
| --- | --- | --- | --- | --- | --- | --- | --- |
| **512** | **1** |  |  |  |  |  |  |
| **128** | **1.73E-03** | **1** |  |  |  |  |  |
| **32** | **6.89E-21** | **6.59E-07** | **1** |  |  |  |  |
| **16** | **2.76E-17** | **2.72E-11** | **1.02E-14** | **1** |  |  |  |
| **8** | **1.60E-209** | **1.79E-224** | **7.16E-253** | **5.72E-178** | **1** |  |  |
| **4** | **< 2.2E-16** | **< 2.2E-16** | **< 2.2E-16** | **< 2.2E-16** | **4.72E-198** | **1** |  |
| **2** | **< 2.2E-16** | **< 2.2E-16** | **< 2.2E-16** | **< 2.2E-16** | **< 2.2E-16** | **3.77E-122** | **1** |

P-values were calculated using the Chi-squared test.

At a Bonferroni-corrected significance level of p < 0.00238, all distributions by each MSA depth were statistically different from each other.

**^a^** MSA Depth corresponds to the number of sequences included in the Multiple Sequence Alignment (MSA) used as input for AF2 through ColabFold.
