# Supplementary material for: A comprehensive exploration of the druggable conformational space of protein kinases using AI-predicted structures
Source: PLoS Comput Biol. 2024 Jul 24;20(7):e1012302. doi: 10.1371/journal.pcbi.1012302 (PMC11268620; doi:10.1371/journal.pcbi.1012302)
Supplement: S3 Table — P-values were calculated using the one-sided Wilcoxon rank-sum test at a significance level of 0.05. a Group indicates kinase group within which comparisons are being made. b ‘CIDI’ indicates the significance of the p(AF2CIDI > 8MSACIDI) comparison of ‘CIDI’ fractions. All other comparisons indicate comparison by p(AF2CONF < 8MSACONF). (DOCX) [file pcbi.1012302.s010.docx]

**S3 Table. Comparison of conformation fractions of kinase models deposited in AF2 Database and predicted by AF2 at MSA depth 8.**

| **Group^a^** | **CIDI^b^** | **CIDO** | **CODI** | **CODO** | **DFGinter** | **Unassigned** |
| --- | --- | --- | --- | --- | --- | --- |
| **AGC** | **< 2.2e-16** | **< 2.2e-16** | **1** | **1** | **< 2.2e-16** | **< 2.2e-16** |
| **CAMK** | **< 2.2e-16** | **< 2.2e-16** | **1** | **< 2.2e-16** | **< 2.2e-16** | **< 2.2e-16** |
| **CK1** | **< 2.2e-16** | **< 2.2e-16** | **< 2.2e-16** | **< 2.2e-16** | **< 2.2e-16** | **< 2.2e-16** |
| **CMGC** | **< 2.2e-16** | **< 2.2e-16** | **1** | **< 2.2e-16** | **< 2.2e-16** | **< 2.2e-16** |
| **Other** | **< 2.2e-16** | **1** | **1** | **< 2.2e-16** | **< 2.2e-16** | **< 2.2e-16** |
| **STE** | **< 2.2e-16** | **< 2.2e-16** | **1** | **< 2.2e-16** | **< 2.2e-16** | **< 2.2e-16** |
| **TK** | **< 2.2e-16** | **1** | **1** | **< 2.2e-16** | **1** | **< 2.2e-16** |
| **TKL** | **< 2.2e-16** | **1** | **1** | **< 2.2e-16** | **< 2.2e-16** | **< 2.2e-16** |

P-values were calculated using the one-sided Wilcoxon rank-sum test at a significance level of 0.05.

^a^ Group indicates the kinase group within which comparisons are being made.

^b^ ‘CIDI’ indicates the significance of the p(AF2_CIDI_ > 8MSA_CIDI_) comparison of ‘CIDI’ fractions. All other comparisons indicate comparison by p(AF2_CONF_ < 8MSA_CONF_).
