# Supplementary material for: A comprehensive exploration of the druggable conformational space of protein kinases using AI-predicted structures
Source: PLoS Comput Biol. 2024 Jul 24;20(7):e1012302. doi: 10.1371/journal.pcbi.1012302 (PMC11268620; doi:10.1371/journal.pcbi.1012302)
Supplement: S4 Table — a Name of kinase in enrichment analysis. b Average area-under-the-curve (AUC) from enrichment plots of all of each kinase’s AF2 models. c Standard deviation of the distribution of enrichment AUCs for each kinase’s AF2 models. d Highest AUC of all enrichment plots for each kinase’s AF2 models. (DOCX) [file pcbi.1012302.s011.docx]

**S4 Table. Average and max AUCs for enrichment of AF2 kinase models.**

| **Kinase^a^** | **avgAUC^b^** | **Standard Dev.^c^** | **maxAUC^d^** |
| --- | --- | --- | --- |
| **ABL1** | **63.40** | **7.69** | **71.95** |
| **AKT2** | **70.06** | **18.79** | **86.27** |
| **BRAF** | **61.47** | **5.20** | **73.34** |
| **CDK2** | **76.58** | **7.82** | **93.09** |
| **CSF1R** | **55.42** | **5.24** | **67.77** |
| **EGFR** | **67.44** | **6.46** | **76.72** |
| **FGFR1** | **55.19** | **11.35** | **76.09** |
| **IGF1R** | **75.34** | **3.70** | **80.67** |
| **JAK2-1** | **64.96** | **10.93** | **88.40** |
| **JAK2-2** | **80.16** | **4.17** | **89.51** |
| **KDR** | **64.80** | **8.81** | **80.75** |
| **KIT** | **66.81** | **14.32** | **92.64** |
| **LCK** | **61.23** | **10.01** | **82.26** |
| **MAPK10** | **62.49** | **20.64** | **89.68** |
| **MAPK14** | **39.48** | **6.89** | **53.80** |
| **MAPK1** | **62.06** | **5.93** | **75.79** |
| **MAPKAPK2** | **56.67** | **20.48** | **71.72** |
| **MET** | **66.75** | **10.05** | **79.98** |
| **PLK1** | **58.11** | **19.51** | **70.72** |
| **PTK2** | **79.28** | **6.98** | **93.67** |
| **ROCK1** | **73.71** | **21.59** | **83.51** |
| **SRC** | **53.35** | **16.48** | **70.09** |
| **TGFBR1** | **62.78** | **32.13** | **87.71** |
| **WEE1** | **71.41** | **26.55** | **88.28** |

**^a^** Name of kinase tested in enrichment analysis

**^b^** Average area-under-the-curve (AUC) from enrichment plots of all of each kinase’s AF2 models

**^c^** Standard deviation of the distribution of enrichment AUCs for each kinase’s AF2 models

**^d^** Highest AUC among all enrichment plots for each kinase’s AF2 models
